# Unfinished nursing care in healthcare settings during the COVID-19 pandemic: a systematic review

**DOI:** 10.1186/s12913-024-10708-7

**Published:** 2024-03-19

**Authors:** Aysun Bayram, Stefania Chiappinotto, Alvisa Palese

**Affiliations:** 1 Faculty of Health Sciences, Karadeniz Technical University, Trabzon, Turkey; 2 Department of Medicine, University of Udine, Udine, Italy

**Keywords:** Unfinished nursing care, COVID-19 pandemic, Reasons, Consequences

## Abstract

**Background:**

Unfinished nursing care is becoming increasingly more of a concern in worldwide healthcare settings. Given their negative outcomes, it is crucial to continuously assess those nursing interventions that are commonly postponed or missed, as well as the underlying reasons and consequences. The worldwide COVID-19 pandemic has made it difficult for health facilities to maintain their sustainability and continuity of care, which has also influenced the unfinished nursing care phenomenon. However, no summary of the studies conducted during the COVID-19 pandemic was produced up to now. The main aim of this study was to systematically review the occurrence of, reasons for, and consequences of unfinished nursing care among patients in healthcare settings during the COVID-19 pandemic.

**Methods:**

Systematic review registered in PROSPERO (CRD42023422871). The Preferred Reporting Items for Systematic Reviews and Meta-Analyses statement guideline and the Joanna Briggs Institute Critical Appraisal tool for cross-sectional studies were used. MEDLINE-PubMed, the Cumulative Index to Nursing and Allied Health Literature, and Scopus were searched from March 2020 up to May 2023, using keywords established in the field as missed care, unfinished nursing care, or implicit rationing.

**Results:**

Twenty-five studies conducted mainly in European and Asiatic countries were included and assessed as possessing good methodological quality. The following tools were used: the MISSCARE Survey (= 14); the Basel Extent of Rationing of Nursing Care (= 1), also in its revised form (= 2) and regarding nursing homes (= 2); the Perceived Implicit Rationing of Nursing Care (= 4); the Intensive Care Unit-Omitted Nursing Care (= 1); and the Unfinished Nursing Care Survey (= 1). The order of unfinished nursing care interventions that emerged across studies for some countries is substantially in line with pre-pandemic data (e.g., oral care, ambulation). However, some interesting variations emerged at the country and inter-country levels. Conversely, labour resources and reasons close to the emotional state and well-being of nurses were mentioned homogeneously as most affecting unfinished nursing care during the pandemic. None of the studies investigated the consequences of unfinished nursing care.

**Conclusions:**

Two continents led the research in this field during the pandemic: Europe, where this research was already well established, and Asia, where this research is substantially new. While unfinished care occurrence seems to be based on pre-established patterns across Europe (e.g., regarding fundamentals needs), new patterns emerged across Asiatic countries. Among the reasons, homogeneity in the findings emerged all in line with those documented in the pre-pandemic era.

**Supplementary Information:**

The online version contains supplementary material available at 10.1186/s12913-024-10708-7.

## Background

Unfinished nursing care (UNC), which is becoming increasingly more of a concern in worldwide healthcare settings, involves the skipped, delayed, or incomplete delivery of nursing interventions needed for the patient and/or the patient’s family [1, 2]. The prevalence of UNC, which ranges from 55 to 98% globally [1], is considered as an accurate indicator of both patient safety and nursing care quality [3, 4]. The primary reasons for UNC are issues in communication, labour, and material resources [5]. The occurrence of UNC has also been associated with staff shortage and factors at both the structural level (e.g., nurses’ roles and experiences) and the process level, such as the stressful work environment, some negative managerial practices, the amount of overtime, and the high and/or complex demand for patient care [6–11]. In terms of consequences, UNC is linked to poor patient (e.g., pressure sores), nurse (e.g., moral distress), and organisational outcomes (e.g., increased length of stay) [5, 12–14]. Given these unfavourable outcomes, it is crucial to continuously assess those nursing interventions that are commonly postponed or missed, as well as the underlying reasons and consequences, to inform evidence-based strategies aimed at decreasing the frequency of UNC.

The worldwide COVID-19 pandemic has made it difficult for health facilities to maintain their sustainability and continuity of care due to the dramatic call to increase the care capacity with limited resources [15–17]. The staff sector most impacted by the pandemic — especially due to concerns regarding infection — has been recognised as nursing staff delivering direct patient care and thus representing the most crucial element of the health system infrastructure [18]. In addition to the need to increase the amount of care, nurses have also been impacted by unfamiliar work settings due to changes in the layout of the hospitals, sickness exposure, and urgent deployment from one department to another without the required skills. Therefore, various components (e.g., communication) of nursing care have been compromised by the limited interaction required during the pandemic and the need to be distanced. Nurses’ care capacity has also been negatively impacted by feelings related to the pandemic triggering anxiety, depression, and burnout [19, 20]. A rise in the number of nurses layoffs, the increased shortage of nurses, poor working circumstances, negative feelings, and imbalances in the nurse–patient ratio may all have increased the occurrence of UNC during the pandemic [21, 22] by further eroding the quality of care [23, 24]. Gurkovà et al. [25] stated that UNC may have increased the risk and adverse effects of the COVID-19 pandemic, resulting in ethical issues and a widespread mistrust in health systems [26]; moreover, Nash et al. [27] also stated that healthcare disparities were the consequences of UNC.

However, while the pre-pandemic occurrence of UNC has been well established, with several primary studies and systematic reviews (e.g., [28]) also investigating the underlying reasons (e.g., [29]), no summary of the studies conducted during the pandemic has been provided to date. Summarising the evidence produced may highlight the issues experienced during the pandemic in order to prevent them in future epidemiological disasters. It may also provide information on the quality of care in dramatic circumstances and the variations, if any, in the routine care before the pandemic. Finally, it may also set a new baseline in the context of UNC given the profound disruption and changes affecting the healthcare systems, requiring a long-term recovery. Thus, the aim of this review was to systematically review the occurrence of, reasons for, and consequences of UNC among patients in healthcare settings in the face of the COVID-19 pandemic.

## Methods

### Design

To begin with, two researchers (AB, SC) performed a rapid literature search to establish whether any studies had been published on UNC occurrences, their reasons, and consequences among patients during the pandemic. The beginning of the pandemic period was defined as 11 March 2020, according to the declaration by the World Health Organisation [30].

According to the Population (P), Exposure (E), Comparator (C), Outcomes (O), and Study Design (S) framework [31], the following were considered: P, patients in any healthcare setting; E, the COVID-19 pandemic period, as started on 11 March 2020 up to 5 May 2023 [30]; C, none; O, occurrence, reasons, and consequences of UNC, as perceived by nursing staff; and S, any types of quantitative study designs. Consequently, the following research questions were identified: (1) What was the occurrence of the UNC phenomenon among patients during the pandemic? (2) What were the reasons for the UNC during the pandemic? (3) What were the consequences of the UNC among patients during the pandemic? (4) What were the main methodological features of studies designed/conducted during the pandemic?

The systematic review was reported in its methods and findings according to Preferred Reporting Items for Systematic Reviews and Meta Analysis (PRISMA) guidelines [32].

### Ethical considerations

The researchers designed a systematic review protocol that was registered in PROSPERO (CRD42023422871).

### Inclusion and exclusion criteria

Studies were considered if they (1) regarded the nursing field; (2) focused on UNC occurrence, its reasons, and/or consequences during the pandemic, as perceived by nurses and nursing aides; (3) were published in English, Italian, or Turkish; (4) collected the data using a validated tool/instrument in the UNC field; (5) were conducted after 11 March 2020 during the COVID-19 pandemic up to 5 May 2023 [30]; and (6) used any types of quantitative designs (randomised controlled trials, non-randomised controlled trials, cohort studies, prospective or retrospective observational studies, cross-sectional studies, longitudinal studies).

Studies were excluded if they (1) did not address UNC data and/or did not involve nurses/nursing aides or care workers in the nursing field; (2) used non-validated tools/instruments measuring UNC or interviews; (3) were conducted in a paediatric setting, due to its specificity not being comparable with the adult field; (4) were designed as qualitative studies, reviews, commentaries, editorials, or books; (5) were written in other languages; or (6) had an abstract/full text that was not accessible.

### Search method

MEDLINE-PubMed, the Cumulative Index to Nursing and Allied Health Literature (CINAHL), and Scopus were searched to identify the eligible studies as sources on 5 May 2023. According to the uniqueness of this research, where no MeSH terms have been established and different key words are used [1, 2], all synonymous and equivalent keywords established in the field of UNC were used to access the databases. Specifically, the following keywords were used: “nurse”, “nursing”, “missed care”, “missed nursing care”, “unfinished nursing care”, “unfinished care”, “implicit rationing of nursing care”, “implicit rationing”, “rationing of nursing care”, “rationed care”, “prioritization process”, “omitted nursing care”, “task left undone”, and “task undone” using “OR” and “AND” operators (Supplementary Table 1).

### Quality Appraisal

The Joanna Briggs Quality Appraisal Tool for analytical cross-sectional studies was used in the quality assessment for all eligible studies when they were based on cross-sectional designs [33]. This tool contains eight items with response options of yes, no, unclear, and not applicable. These items regarded inclusion criteria, subjects and setting description, exposure, standard criteria for measurement of the condition, confounding factors, strategies to deal with confounding factors, outcomes measurement, and statistical analysis. Two researchers (AB, SC) independently assessed the quality of the studies as “Rater 1” and “Rater 2”. In the case of a disagreement, the senior researcher (AP) was consulted to reach a consensus, as summarised analytically in Supplementary Table 2.

Besides the quality appraisal, to prevent bias, the following strategies were applied: (a) all researchers contributed to the writing of the review protocol; (b) at least two researchers searched the literature, chose the studies, and extracted the data, independently; (c) the senior researcher oversaw the data extraction; and (d) agreement was required before moving on to each next step.

### Data extraction and synthesis

All studies that met the inclusion criteria, regardless of the results of their methodological quality, underwent the data extraction and data synthesis. The studies were divided into two groups and shared between two researchers (AB, SC). *In primis*, the data extraction grid was piloted in one study, and the findings agreed: no changes were required. Then, researchers independently extracted data from the remaining studies by populating the grid with the following data: (1) author(s), year, and country; (2) study aim(s) and design; (3) sample and setting; and (4) period of data collection and tool(s). Then the findings of the quality appraisal were provided (Table 1). At the end of data extraction, the researchers rechecked the data. Disagreements were solved with the consultation of the senior researcher (AP) until consensus was reached.

**Table 1:** Main characteristics of the included studies (= 25)

Author(s) Year Country	Aim(s) & Study Design	Sample & Setting	Period of Data Collection & Tools	Quality Appraisal^a^ Y/N/U/NA
Albsoul et al. [34] 2022 Jordan	To identify the perceptions of nurses for UNC and the reasons for UNC across three health-care sectors: public, private and university Cross-sectional survey	672 questionnaires were completed by registered nurses working in medical and surgical wards 10 acute-care hospitals	March–July 2021 MISSCARE survey	6/1/0/1
Alfuqaha et al. [35] 2022 Jordan	To compare perception of nurses about UNC for patients before and during the COVID-19; to examine how nurses differed in terms of the type of UNC and the factors that contributed to it before and during the COVID-19 pandemic Comparative cross-sectional study	260 nurses Medical/ surgical wards and intensive care units of a tertiary hospital	From November 2019 to May 2020 MISSCARE survey – Arabic version	6/0/1/1
Al Muharraq et al. [36] 2022 Saudi Arabia	To explore the dimensions of UNC and its predictors Cross-sectional study	604 staff nurses Inpatient wards in the Jazan area (2 tertiary and 8 general hospitals)	June-September 2021 MISSCARE survey	7/0/0/1
Cengia et al. [37] 2021 Italy	To compare the occurrence and the reasons for UNC among COVID-19 and non-COVID-19 patients as perceived by nurses Comparative cross-sectional study	479 registered nurses 22 units (15 COVID-19 and 7 non-COVID-19 units) caring for medical, geriatric, medical-surgical, and orthopaedic patients	November 2020–January 2021 The Unfinished Nursing Care Survey	5/2/0/1
Falk et al. [38] 2022 Sweden	To describe and evaluate reported UNC in the critical care context before and during different phases of the COVID-19 pandemic Comparative cross-sectional study	134 nurses Critical care units at a university hospital	*First period*: October 2019 *Second period*: November 2020 *Third period*: May 2021 MISSCARE survey – Swedish version	6/0/1/1
Gurková et al. [25] 2021 Czech Republic	To examine the differences in reasons for UNC according to the type of hospitals and wards; to determine the relationship between the reasons for UNC and job satisfaction Cross-sectional correlational study	371 nurses 4 hospitals: 1 university and 3 regional hospitals (internal medicine and surgical areas in a region)	May–September 2020 MISSCARE survey – Czech version	7/0/0/1
Gurková et al. [39] 2022 Czech Republic	To examine the frequencies, type of UNC, and the associations between nurses’ reported nurse work environment and UNC variables during the COVID-19 pandemic at inpatient medical and surgical wards Observational cross-sectional study	371 nurses 30 inpatient wards of four acute care hospitals	April–September 2020 MISSCARE survey	6/1/0/1
Hackman et al. [40] 2023 Finland	To describe UNC activities in nursing homes Descriptive cross-sectional study	2700 care workers 69 nursing homes representing four public organizations	January–May 2021 BERNCA-NH	6/0/1/1
Hosseini et al. [41] 2022 Iran	To investigate UNC and its reasons during the COVID-19 pandemic Cross-sectional study	135 nurses COVID-19 units at educational hospitals	Summer 2020 MISSCARE survey – Persian version	5/0/2/1
Jarosz et al. [42] 2022 Poland	To assess the level of rationing care, fatigue, job satisfaction and occupational burnout and the relationship between them Cross-sectional study	130 nurses Urology departments at a hospital	March–May 2021 PIRNCA	6/0/1/1
Jarosz & Mlynarska [43] 2023 Poland	To assess the impact of place of residence, forms of postgraduate education, work system, number of patients per one nurse on duty, satisfaction with the financial situation, number of diseases the nurse suffers from on the rationing of nursing care in urology wards Cross-sectional study	130 nurses Urology departments at a hospital	March–May 2021 PIRNCA	6/0/1/1
Khrais et al. [44] 2022 Jordan	To examine the relationship between UNC and perceived organizational support, accountability and nurses’ characteristics under the impact of COVID-19 Cross-sectional study	536 nurses Three public hospitals, three private hospitals and the two teaching hospitals	March–May 2021 MISSCARE survey	7/0/0/1
Labrague et al. [45] 2022 Sultanate of Oman	To examine UNC, overall quality of nursing care, and factors that may influence nurses’ intent to omit or complete required nursing tasks during the pandemic Cross-sectional study	295 clinical frontline nurses 14 hospitals (seven government and seven private hospitals)	November–December 2020 MISSCARE survey	7/0/0/1
Maghsoud et al. [46] 2022 Iran	To investigate the mediating role of implicit rationing of nursing care, job satisfaction and emotional exhaustion in the relationship between workload and quality of nursing care Cross-sectional study	311 nurses 4 different hospitals	October–December 2020 BERNCA	6/0/1/1
Mingude et al. [47] 2022 Ethiopia	To assess the magnitude, reason and associated factors of UNC Cross-sectional study	315 nurses Medical, paediatric, surgical and gynaecology wards in 7 hospitals	April 2021 MISSCARE survey	7/0/0/1
Nymark et al. [48] 2021 Sweden	To evaluate UNC and patient safety during the outbreak and first wave of the COVID-19 pandemic at the in-patient wards at the cardiology department Cross-sectional study with a comparative approach	43 registered nurses and nursing assistants Cardiology department (two highly specialized medical wards and two intensive coronary care units)	May–June 2020 MISSCARE survey – Swedish version	5/0/2/1
Rahmani et al. [49] 2021 Iran	To evaluate UNC and its relationship with nurses’ patient safety attitudes at hospitals Observational correlational study	351 nurses 9 Tabriz University of Medical Sciences hospitals	2021 MISSCARE survey – Persian version	7/0/0/1
Schneider-Matyka et al. [50] 2023 Poland	To assess the effect of stress on rationing of nursing care Observational cross-sectional study	800 nurses, 567 of whom participated Primary health-care facility; county hospital; teaching hospital; regional hospital	From September 2020 to December 2021 PIRNCA	6/0/1/1
Tomaszewska et al. [51] 2021 Poland	To assess the rationing of the level of nursing care among nurses employed at a district hospital Cross-sectional study	295 nurses District hospital	September–December 2020 BERNCA-R – Polish version	5/0/2/1
Uchmanowicz et al. [52] 2021 Poland	To assess the relationship between the rationing of nursing care and professional burnout in nursing staff Cross-sectional design	219 cardiac nurses Non-invasive cardiology wards of four hospitals in Wroclaw	January–May 2020 BERNCA-R – Polish version	6/0/1/1
Vincelette et al. [53] 2022 Canada	To describe the characteristics of omitted nursing care in ICU; to examine the relationship between work environment features, omitted nursing care and nurse-reported outcomes in the ICU Cross-sectional correlational study	2107 ICU nurses from Quebec’s Board of Nurses, 564 ICU nurses participated ICU units	Over September 2021 The ICU-ONC	7/0/0/1
von Vogelsan et al. [54] 2021 Sweden	To determine frequencies, types of and reasons for UNC during the COVID- 19 pandemic at inpatient wards Comparative cross-sectional study	130 registered nurses and nursing assistants in pandemic (COVID-19 sample) 157 registered nurses and nursing assistants in pre-pandemic (reference sample) A highly specialized university hospital in medical/surgical departments	May–June 2020 (COVID-19 sample) October 2019 (reference sample) MISSCARE survey – Swedish version	6/0/1/1
Xie et al. [55] 2023 China	To examine the effect of role overload, work addiction and leader–member exchange on UNC Cross-sectional study	420 RNs, 403 of whom participated One general tertiary hospital from five cities in five regions, randomly selected	March–May 2022 MISSCARE survey	7/0/0/1
Yuwanto et al. [56] 2023 Indonesia	To assess the Indonesian version of the PIRNCA instrument to psychometric properties Descriptive cross-sectional study	214 RNs working in inpatient units, 167 of whom participated Two government hospitals	May 2021 PIRNCA	5/1/1/1
Zhang et al. [22] 2021 China	To provide initial evidence on implicit rationing of nursing care in publicly funded nursing homes in Shanghai with a particular focus on the association between care workers’ training needs and implicit care rationing Cross-sectional study	374 care workers Publicly funded nursing homes in Shanghai	September–November 2020 BERNCA-NH	7/0/0/1

**Legend:** BERNCA: Basel Extent of Rationing of Nursing Care; BERNCA-NH: Basel Extent of Rationing of Nursing Care for Nursing Homes; BERNCA-R: Revised Basel Extent of Rationing of Nursing Care-Revised; COVID-19: Coronavirus-19; ICU: Intensive Care Unit; ICU-ONC: the Intensive Care Unit Omitted Nursing Care instrument; PIRNCA: perceived Implicit Rationing of Nursing Care; RN: Registered Nurse; UNC: unfinished nursing care; Y: Yes; N: No; U: Unclear; NA: Not Applicable

^a^ the first number indicates the number of ‘Yes’ answers; the second the numberof ‘No’ answers; the third the number of ‘Unclear’ answers; the fourth the number of ‘Not available’ answers

A narrative synthesis process was used to summarise the findings [57] according to the review questions, applying the following methodology:

1. Studies conducted during the pandemic and their methodological quality: the researchers conducted a preliminary synthesis to provide an initial description of the main characteristics of the studies and their methodological quality, and similarities and differences across studies were presented by using textual explanations [57].
2. The occurrence of UNC: Findings were tabulated according to the tools used in each study, namely the MISSCARE Survey, the Basel Extent of Rationing of Nursing Care (BERNCA) and the Revised BERNCA (BERNCA-R), the Perceived Implicit Rationing of Nursing Care (PIRNCA), the Basel Extent of Rationing of Nursing Care for Nursing Homes (BERNCA-NH), the Intensive Care Unit Omitted Nursing Care instrument (ICU-ONC), and the Unfinished Nursing Care Survey (UNCS). In all tools, participants are required to rank the nursing interventions missed and/or postponed from always to never. Then, according to the following considerations,

- the tools used different metrics (Likert from 1 to 5 for MISSCARE Survey and UNCS, from 0 to 4 for BERNCA, from 0 to 3 for PIRNCA, from 1 to 4 for BERNCA-NH, from 1 to 4 for ICU-ONC) and differed in the direction of measures (e.g., from always missed to never missed, e.g., [43], or the opposite, e.g. [50]); and
- UNC interventions reflect an order [58, 59], such as first, second, and third, of interventions missed, expressing a prioritisation process (what should be actualised first and what can be delayed).

Data regarding the position (= order) of each nursing intervention according to the averages documented in the studies were extracted and then ranked according to the position: for example, the average of 3.23 with the MISSCARE Survey [35], indicating that this was the most unfinished activity, was ranked as first. Then, according to Blackman and colleagues [60], the first three interventions of high occurrence of being unfinished were identified; from the fourth to the sixth, those of intermediate occurrence; and from the seventh to ninth, those of a low occurrence of UNC.

(3) The UNC reasons: Reasons were summarised based on the following considerations:

- Studies using the MISSCARE Survey and the UNCS reported the reasons for UNC item by item, according to the structure of the tool;
- Other studies documented the relationships (as correlations, associations) indicating a significant role of some factors in increasing/hindering UNC during the pandemic.

In the first case, the reasons were extracted and analysed in the same manner as UNC activities; in the second, studies (22 out of 25) documenting a statistically significant relationship of given factors with the UNC were extracted and categorised as organisational, work, or individual factors according to the literature in the field [29]. Of the remaining three studies, which were not focused on the reason for UNC, one was a methodological study that was focused on the psychometric assessment of the tool [56], one was a comparative study that was focused on the comparison between the data from a COVID-19 sample and a reference sample [54], and one was a study in which conditions were identified affected by the consequences of UNC [48].

(4) UNC main consequences: if any, were described narratively.

All researchers were involved in the data analysis and synthesis process to ensure rigour in the process.

## Findings

The results regarding the included studies are described below, including an exploration of their characteristics and quality and the occurrence of, reasons for, and consequences of UNC.

### Search outcomes

In total, 1,389 articles were identified from the electronic databases. The search results were transferred to a reference manager (Mendeley) to organise the data extraction process. First, three steps were followed for the study selection: in the first stage, titles, in the second stage, abstracts, and in the third stage full text of the retrieved studies were screened for their eligibility by two reviewers (AB, SC), independently. In the case of any disagreement, the opinions of a third senior researcher (AP) were consulted during the entire process. Consensus between the researchers was essential for study inclusion.

**Fig. 1:**
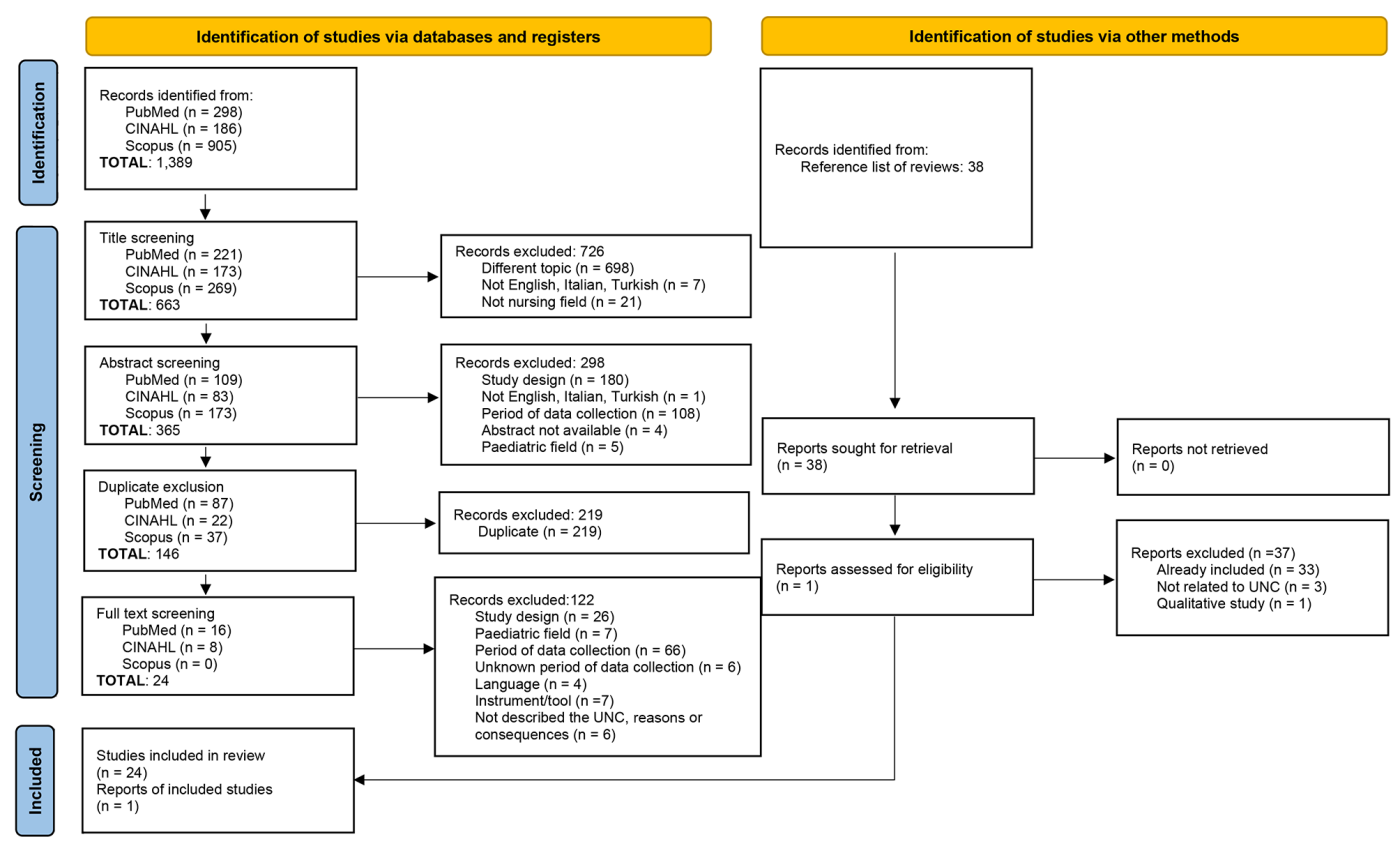
PRISMA flow chart

In the first stage, 726 studies were excluded; from 1,389 studies, 663 articles were retained for abstract screening. Thus, in the second stage, 298 studies were excluded. At this stage, 365 studies met the criteria for next-step screening. Before the full-text screening, 219 duplicated studies were removed, and a visual inspection was conducted by two researchers (AB, SC) to check for duplicates. Then, 146 studies remained for full-text screening, and 122 of them were excluded for different reasons, as reported in Fig. 1. The references of the excluded reviews were screened by two researchers (AB, SC) to check their eligibility in an independent fashion and then agreed upon. In total, 38 articles were checked, of which 33 were already included, three were not related to UNC, and one was a qualitative study design. At the end of the screening process, 25 studies were included (Fig. 1).

### Included studies and their quality

Out of the 25 studies included (Table 1), 20 used a descriptive cross-sectional design (e.g., [34]) and five a comparative cross-sectional design confronting the data (a) before and during the pandemic [35]; (b) or before the pandemic, and the second/third wave [38]; and (c) of the COVID-19 sample and the reference sample [37, 48, 54]. Most studies were conducted in Europe (= 12, e.g., [50]) and Asia (= 11, e.g., [45]). Of the remaining, one was carried out in Africa [47] and one in Canada [53]. Study locations ranged from a hospital (e.g., [35]) to specific hospital settings (tertiary [55], district [51], government [56], private [34], teaching [50]) in various types of units (e.g., medical/surgical [54], urology [43], cardiology [48]). In addition, COVID-19 units were included in two studies [22, 37, 41] and nursing homes in another two [22, 40].

Studies were published between 2020 and 2023; however, nine of them completed the data collection in 2020 (e.g., [52]), 10 in 2021 (e.g., [47]), two between 2020 and 2021 [37, 50], one in 2022 [55], two between 2019 and 2020 [35, 54], and one between 2019 and 2021 [38]. Participants were mainly nurses, and their sample size ranged from 130 [42] to 672 [34] in 21 studies; in others, participants were generally identified as “care workers”, ranging from 374 [22] to 2,700 [40], while those including nursing assistants and registered nurses together ranged from 43 [48] to 287 [54] participants. The MISSCARE Survey tool was the most used (= 14, e.g., [44]), followed by BERNCA (= 1, [46]), Revised BERNCA (BERNCA-R) (= 2, [51, 52]), BERNCA-NH (= 2, [22, 40]), PIRNCA (= 4, e.g., [42]), ICU-ONC (= 1, [53]), and UNCS (= 1, [37]) (Table 1).

All studies reported a good methodological quality with minimal bias (Supplementary Table 2). Most were ranked positively for at least six (“yes” responses) out of eight questions (= 11; e.g., [39]), nine studies for at least seven questions (e.g., [44]), and five for at least five questions (e.g., [41]). Four studies failed to clarify the strategies to deal with confounding factors (e.g., [56]), while seven described these strategies unclearly (e.g., [51]). The settings and study subjects were stated as being unclear in eight studies (e.g., [52]). Additionally, in one study, the sample inclusion criteria were not detailed, while in another study, the confounding factors were not reported. The objective, standard criteria used to measure the condition were not assessable in any of the qualified studies, since the condition was considered the COVID-19 disease. At the overall level, all except six studies [25, 34, 42, 43, 46, 55] documented the occurrence of and reasons for UNC activities.

### The occurrence of UNC

In the 14 studies based on the MISSCARE survey, the most frequent UNC activities were “Ambulation 3 times per day or as ordered”, “Turning patient every two hours”, “Attending interdisciplinary care conferences whenever held”, “Providing mouth care”, and “Patient teaching about procedures, tests and other diagnostic studies”. In particular, “Ambulation 3 times per day or as ordered” was the activity most missed in three studies [35, 38, 39]; it was the second unfinished activity in the study by Al Muharraq et al. [36] and the third in another three studies ([48]; in both the COVID-19 sample and the reference sample of von Vogelsang et al. [54]) (Table 2, Supplementary Table 3). “Turning patient every two hours” was the most frequent UNC activity in two studies (in the COVID-19 sample of Nymark et al. [48]; in the reference sample of von Vogelsang et al. [54]) and the second in another three ([35]; in the reference sample of Nymark et al. [48]; in the third wave sample of Falk et al. [38]). This activity was third in another four studies ([35, 36, 38]; second wave [47]) (Table 2, Supplementary Table 3). However, the first unfinished activity in five studies was “Attending interdisciplinary care conferences whenever held” ([36, 44, 49]; in the reference sample of Nymark et al. [48]; in the COVID-19 sample of von Vogelsang et al. [54]) and “Monitoring patient” in one study [45] (Table 2, Supplementary Table 3). Conversely, the least frequently unfinished activities were “Monitoring intake/output”, “Vital signs assessed as ordered”, “Bedside glucose monitoring”, and “Patient assessments every shift” (Table 2, Supplementary Table 3).

**Table 2:** The occurrence of and reasons for unfinished nursing care in studies using the MISSCARE survey [59]

Interventions		Albsoul et al. [34] ^a^	Alfuqaha et al. [35]		Al Muharraq et al. [36]	Falk et al. [38]			Gurkova et al. [25]^b^	Gurková et al. [39]	Hosseini et al. [41]	Khrais et al. [44]	Labrague et al. [45]^c^	Mingude et al. [47]	Nymark et al. [48]		Rahmani et al. [49]^d^	von Vogelsan et al. [54]		Xie et al. [55]^e^
PART A, Interventions																				
			**B**	**D**		**B**	**W** ^**2**^	**W** ^**3**^							**CS**	**RS**		**CS**	**RS**	
Ambulation 3 times per day or as ordered			•••	•••	•••	•••	••	••		•••		••	••		•••	••	•	•••	•••	
Assess effectiveness of medications			•							•							•	••	•	
Turning patient every 2 h			•••	•••	•••	••	•••	•••		••				•••	•••	•••	••	•••	•••	
Mouth care			•••	••	••			•		•		•	••		••	•••	•••	••	••	
Patient teaching about procedures, tests and other diagnostic studies			••	••	••	•	•			••	•••	••					•			
PRN medication requests acted on within 15 min														•••	•					
Full documentation of all necessary data										••	••	•••		•						
Feeding patient while the food is still warm				••	••	•••	•••	•••			•••					••	••		•	
Medications administered within 30 min before or after scheduled time								•								••			•	
Assist with toileting needs within 5 min of request				•	•	••	•	•		•							•••			
Response to call light is initiated within 5 min						•						•••								
Emotional support to patient and/or family			••	•••	•		•			•••	•••		•••			•	•	•	••	
Patient bathing/skin care			•		•										•		•••			
IV/central line site care and assessments according to hospital policy														••	•		•	•		
Teach patient about plans for their care after discharge						•••	••	••				••	•		••	•		••	••	
Monitoring intake/output											••		•••	•••			•			
Setting up meals for patients who feed themselves			•			•••	•••	•••								•	•••			
Vital signs assessed as ordered														••			•			
Focused reassessments according to patient condition				•									••	•			••			
Hand-washing											••	•					•			
Bedside glucose monitoring as ordered												•		•						
Patient assessments performed each shift																	•			
r-Attend interdisciplinary care conferences whenever held			••	•	•••	••	••	••				•••		••	•••	•••	•••	•••	•••	
r- Skin/wound care						•		•					•••		••			•		
r-Interdisciplinary care conferences whenever held										•••			•							
r-Discussing patient expectation														•••						
r-pain assesment and management														•						
r-Physical examination																				
r-Initial assesment																				
r-Review of collected lab results																				
Treatments and procedures													•							
PART B, Reasons																				
Communication	Tension or communication breakdowns within the nursing team					•	•	•	•••			•••			•	•		•	•	
	Lack of backup support from team members				•	•	•	•	••			•••			••	•		••	•	
	Nursing assistant did not communicate that care was not done						•	•	•		••	•		•	•	•		•	••	
	Caregiver is off unit or unavailable					•	••	••	••											
	Tension or communication breakdown with the medical staff						•	•	•••			••			•	••			•	
	Tension or communication breakdown with other support departments		•				•		•			••								
	Other departments did not provide the care needed							•	••											
	Inadequate hand-off from previous shift or sending unit			••					••			•								
	Unbalanced patient assignments		•		••	••	••	••	••		•••	•••			••	••		••	••	
	r-Inadequate supervision of nursing assistants																			
	r-Lack of cues/reminders																			
Material resources	Supplies/equipment not available when needed	•	•	••	•				•••		••			•••						
	Supplies/equipment not functioning properly when needed	•	••	•	••				••		••			••						
	Medications were not available when needed	•		•••		••	•		•••			•		••				•		
Labor resources	Unexpected rise in patient volume and/or acuity on the unit	•••	••	••	•••	•••	•••	•••	••					••	•••	•••		•••	•••	
	Urgent patient situations (e.g. a patient’s condition worsening)		••	•••	•	•••	•••	•••	••		•••	••		•••	•••	•••		•••	•••	
	Inadequate number of staff	•••	•••	•••	•••	•••	•••	•••	•••		•••			•••	•••	•••		•••	•••	
	Inadequate number of assistive personnel (e.g. nursing assistants, technicians, etc.)	•••	•••	•	•••	•	••	•	•••			•		•						
	r-Emotional or physical exhaustion	••																		
	r-Interruptions/Multitasking	••																		
	r-Inadequate support from leadership	••																		
	r-Heavy admission and discharge activity		•••	•	••	••	••	••	•••					•	••	••		••	••	

**Legend:** **, as first, second and third unfinished activities in order according to the statistical values (1st,2nd,3rd); ••, 4th,5th,6th order; •, 7th,8th,9th order; B, before; CS, COVID-19 Sample; D, during; RS, reference sample; W, Wave; a, this study was concentrated on analysing only the elements in Part B in the MISSCARE survey; b, this study defined part B of MISSCARE survey; c, this study defined a few specific items of part A within the main score of the MISSCARE survey; d, this study defined only the results of part A of the MISSCARE survey and overall score of the survey; e, this study defined only the overall score of the MISSCARE survey (see Supp. File 3)

Considering the studies using the PIRNCA tool, the most frequent unfinished interventions were the “Coordination of care and discharge planning” and the least common the “Implementation of prescribed treatment plan” in Schneider-Matyka et al. [50]. Contrarily, Yuwanto et al. [56] discovered that “Coordination of care and discharge planning” were the least frequently unfinished activities. The other most frequent UNC activities were listed in Schneider-Matyka et al. [50] and Yuwanto et al. [56], respectively, as (i) “Offer emotional or psychological support”, (ii) “Converse with team members”, (iii) “Converse with external agency”, and (i) “Routine skin care”, (ii) “Converse with external agency”, and (iii) “Assist with bowel and bladder elimination”, while the least unfinished were, respectively, (i) “Medication administration”, (ii) “Enteral and parenteral nutrition”, and (i) “Converse with patient regarding discharge”, (ii) “Infection control practices” (Table 3, Supplementary Table 4).

**Table 3:** Unfinished nursing care occurrence in studies using the perceived implicit rationing of nursing care [1]

Interventions	Jarosz et al. [42]^a^	Jarosz et al. [43]^a^	Schneider-Matyka et al. [50]	Yuwanto et al. [56]
Assistance with physical care				
Routine hygiene				••
Routine skin care				•••
Change soiled linen				
Assist with ambulation			•	••
Assist with repositioning			•	
Assist with bowel and bladder elimination				•••
Assist with oral nutrition and hydration				••
Promote physical comfort/pain control				
Implementation of prescribed treatment plan				
Medication administration				
Enteral and parenteral nutrition				
Wound care				
Dressing changes				
IV therapy				•
Infection control practices				
Emotional support and teaching				
Teaching patient or family			•	
Prepare patient or family for treatments/procedures				
Offer emotional or psychological support			•••	•
Surveillance/vigilance				
Monitoring physiological status				
Monitoring behaviour				•
Monitoring safety				
Follow-up on status changes/requests/unclear orders				
Timely response to requests			••	
Supervise delegated tasks			••	
Evaluate the plan of care				
Coordination of care and discharge planning				
Converse with team members			•••	
Converse with external agency			•••	•••
Converse with patient regarding discharge			••	
Documentation				
Review documentation			••	
Document initiation/revision of plan of care				
Document assessments and monitoring activities				
Document care/interventions				

**Legend:** **, as first, second and third unfinished activities in order, according to the statistical values (1st,2nd,3rd); 4th,5th,6th order; 7th,8th,9th order; ^a^, this study focused only on the overall score of the tool (see Supp. File 4)

In accordance with Tomaszewska et al. [51] and Uchmanowicz et al. [52], who used BERNCA-R, the most common first, second, and third UNC activities were “Education and training”, “Necessary disinfection measures”, and “Monitoring patients as the nurse felt necessary”. The studies identified “Change of the bed linen”, “Skin care”, and “Assist food intake” as the least frequent UNC activities [51] (Table 4, Supplementary Table 5).

**Table 4:** Ufinished nursing care occurrence in studies based on the basel extent of rationing of nursing care (= 1), revised basel extent of rationing of nursing care (= 2) [61] and basel extent of rationing of nursing care-nursing homes tool (= 2) [62]

BERNCA Interventions	Maghsoud et al. [46]^a^	BERNCA-R Interventions	Tomaszewska et al. [51]	Uchmanowicz et al. [52]^b^	BERNCA-NH Interventions	Hackman et al. [40]	Zhang et al. [22]
1. Activities of daily living (ADLs)		Sponge bath			**Activities of daily living (ADL)**		
(1a) Bathing/skin care		Partial sponge bath			Sponge bath/partial sponge bath/skin care		
(1b) Perform oral or dental hygiene for patients		Skin care			Oral hygiene	•	
(1c) Eating		Oral hygiene			Assist dressing/undressing		
(1d) Mobilization/changing positions		Dental hygiene			Assist food intake		
(1e) Managing body waste (urine, stool, vomit)		Assist food intake			Assist drinking		
(1f) Changing bed linen		Mobilization			Mobilization/change of the position		
2. Caring–Support		Change of the patient’s position			**Caring, Rehabilitation, and Monitoring**		
(2a) Emotional or psychosocial support		Change of the bed linen			Leave a resident in urine/stool longer than 30 min		
(2b) Conversations with patients or their families		Emotional & psychological support		••	Emotional support	•	•••
3. Rehabilitation–Instruction–Education		Necessary conversations			Necessary conversations with resident or family		••
(3a) Toilet training		Information about therapies			Toileting/continence training		
(3b) Activating/rehabilitating care		Continence training (diapers)			Activating or rehabilitating care	•	•••
(3c) Education of patients/their families about self-care		Continence training (insert catheter)			Monitoring residents as care workers feel necessary		
(3d) Preparation for hospital discharge		Activating or rehabilitating care		•••	Monitoring of confused/cognitively impaired residents & use of restraints/sedatives		•
4. Monitoring–Safety		Education and training	•••		Keep residents waiting who rung	••	
(4a) Adequate monitoring of patients’ vital signs		Preparation for discharge			**Documentation**		
(4b) Adequate monitoring of confused/impaired patients		Monitoring patients as described by physician			Studying care plans at the beginning of shift	••	•
(4c) Coping with the delayed response of a physician		Monitoring patients as the nurse feels necessary	•••		Set up or update residents’ care plans	••	••
(4d) Respond promptly to patient calls		Monitoring of confused patients & use of restraints			Documentation of care		
(4e) Adequate hand hygiene		Monitoring of confused patients & use of sedatives			**Social care**		
5. Documentation		Delay in measure because of a physician delay	••		Scheduled single activity with a resident	•••	•
(5a) Review patient documentation at the beginning of the shift		Administration of medication, infusions		••	Scheduled group activity with several residents	•••	•••
(5b) Formulate/update patient care plans		Change of wound dressings	•		Cultural activity for residents with contact outside of nursing home	•••	••
(5c) Documentation of performed nursing care		Preparation for test and therapies	••				
		Keeping patient who has called waiting					
		Adequate hand hygiene	•				
		Necessary disinfection measures	•••				
		Studying care plans		•••			
		Assessment of newly admitted patient	•	•••			
		Set up care plans	••				
		Documentation & evaluation of the care					

**Legend:** ***, as first, second and third unfinished activities in order, according to the statistical values (1st,2nd,3rd); ••, 4th,5th,6th order; •, 7th,8th,9^t^h order; ^a^, this study focused only on the main score of tool; ^b^, this study defined the first five specific items in the results section within the main score of the tool (see Supp. File 5)

In two studies that used the BERNCA-NH tool, “Social care” and “Emotional support” reported the highest occurrences [22, 40]. The most frequent UNC activities were listed in Hackman et al. [40] as (i) “Cultural activity for residents with contact outside of nursing home”, (ii) “Scheduled single activity with a resident”, and (i) “Scheduled group activity with several residents”; in contrast, the most frequent unfinished activities in Zhang et al. [22] were (i) “Activating or rehabilitating care”, (ii) “Emotional support”, and (iii) “Scheduled group activity with several residents”. On the other hand, “Assist dressing/undressing”, “Drinking”, “Food intake”, and “Sponge bath/partial sponge bath/skin care” were listed as the least frequent UNC activities [22, 40] (Table 4, Supplementary Table 5).

In the remaining two studies, recent tools were used. In the study conducted using the ICU-ONC tool, the most common unfinished activities were “Mobilization every two hours”, “Mouth care for intubated patients”, and “Document treatments and procedures”; those least frequent were “Cardiac monitoring surveillance”, “Flag the presence of signs or symptoms of infection”, and “Titrate intravenous perfusions for hemodynamic targets” [53] (Table 5, Supplementary Table 6). In the study using the UNCS [37], the most frequent UNC for both the COVID-19 sample and the reference sample were “Performing bedside glucose monitoring as prescribed”, “Performing clinical handover to adequately inform the next shift nursing team about patients’ conditions”, and “Recording vital signs as planned”, while the least frequently unfinished activities were “Helping patient in need in ambulation”, “Providing passive mobilization/changing position in bedrest patient”, and “Providing mouth care to patients who need it” (Table 6, Supplementary Table 7).

**Table 5:** Unfinished nursing care occurrence in the study using the intensive care unit omitted nursing care instrument [53]

Interventions	Vincelette et al. [53]
Mobilization every two hours	•••
Mouth care for intubated patients	•••
Document treatments and procedures	•••
Timely medication administration	••
Address new prescriptions, consultations	••
Treatment and adverse effects surveillance	••
Venous and arterial catheters care and maintenance	•
Medication-related independent double-check	•
Haemodynamic and physiologic parameters surveillance	•
Draw labs following prescription	
Communicate preoccupations to the medical team	
Neurological signs evaluation	
Pain assessment (patient unable to communicate)	
Provide respiratory care (e.g. aspiration of secretions)	
Sedation adjustment based on prescription (e.g. RASS scale)	
Respond quickly to alarms indicating potential instability	
Pain assessment (patient able to communicate)	
Ensure asepsis in treatments or procedures	
Intervene rapidly to glucose levels (e.g. IV insulin therapy)	
Cardiac monitoring surveillance	
Flag the presence of signs or symptoms of infection	
Titrate intravenous perfusions for haemodynamic targets	

**Legend:** **, as first, second and third unfinished activities in order according to the statistical values (1st,2nd,3rd); ••, 4th,5th,6th order; •, 7th,8th,9th order (see Supp. File 6)

**Table 6:** The occurrence of and reasons for unfinished nursing care in the study using the the unfinished nursing care survey [58]

Interventions	Cengia et al. [37]	
PART A – Interventions	CS	RS
Collect data on the situation of the patients’ care at the beginning of the shift, through the handover	•	••
Perform a round at the beginning of the shift to know the patients, present themselves, and deepen their situation		
Document properly the interventions provided and the revision of the care plan		
Help patient in need in ambulation		
Help patients who need it to get into a chair		
Passive mobilization/changing position in bedrest patient		
Helping patients who are unable to eat independently and/or have clinical problems (e.g. dysphagia)		
Helping patients who are unable to drink independently and/or have clinical problems		
To stimulate the patient to maintain/improve his/her independence		
Provide personal hygiene to patients who need it	••	•
Provide mouth care to patients who need it		
Perform physical assessment (e.g. skin integrity, and invasive device insertion site)		
Check pressure ulcers and change dressing according to protocols	•	
Perform bedside glucose monitoring as prescribed	•••	•••
Monitor intake/output	••	••
Record vital signs as planned	•••	•••
Administer medications within 30 min of the time indicated in the prescription		
Administer PRN medications within 15 min of the patient’s request		
Monitor administered medications effects		
Ensure patients’ comfort (microclimate, patient positioning)		
Monitor pain as planned	•	••
Spend time with patients and their carers		
Communicate with patients and carers		
Inform patients and their carers regarding the nursing care they are receiving		
Emotionally support patients and carers by listening to their needs/concerns		
Involve patients and carers in the discharge planning		
Teach patients and carers how to self-care at home		
Respond promptly to patients’ calls (within 5 min)		
Go to the patients at the bedside without being called		
Ensure intensive surveillance, reevaluating, those patients who are unstable or who present a risk of deteriorating conditions	•	•
Prevent negative outcomes for patients at risk (e.g. falls, pressure ulcers and malnutrition)		
Prevent health-care-associated infections by adopting good clinical practice (e.g. hand hygiene between patients, closed urinary drainage system)	••	••
Discuss with physicians and other staff members the problems of and interventions needed by patients		
Supervise the tasks assigned to the nurse assistants		
Assess the effectiveness of the care provided, for example, reviewing whether nursing care needs have been met		
Fill in/update the clinical documentation/care plan in a comprehensive way		
Perform clinical handover to adequately inform the next shift nursing team about patients’ conditions	•••	•••
Provide clinical teaching to nursing students		•
PART B – Reasons for Unfinished Nursing Care		
Factor 1, Communication		
Tension/conflicts within the nursing staff	•••	•••
Incomplete or interrupted communication among nursing staff	•	•
Tension/conflicts between nursing and medical staff		•
Incomplete or interrupted communication between nursing and medical staff		
Lack of support/collaboration among team members		
Factor 2, Priority setting		
Inadequate nursing care model (e.g. functional task-oriented model of care)	•••	••
Inaccurate initial priority setting	•••	•••
Inadequate priority reassessment during the shift	••	•••
Factor 3, Nurse assistants supervision		
Nurse assistants missed or delayed reporting the tasks left undone	••	•
Inadequate supervision of the tasks assigned to the nurse assistants	••	••
Incomplete or interrupted communication between nursing staff and nurse assistants/assistive personnel	•	••
Factor 4, Material resources		
Medications prescribed not available		
Equipment not available/not functioning properly when needed		
Other departments did not provide the service expected (e.g. delay in diagnostic processes)	•	
Factor 5, Human resources		
Inadequate number of nurses		
Inadequate number of nurse assistants		
Factor 6, Workflow predictability		
Unexpected rise in patient acuity		
Heavy admission/discharge activity during the shift		

**Legend:** **, as first, second and third unfinished activities in order according to the statistical values (1st,2nd,3rd); ••, 4th,5th,6th order; •, 7th,8th,9th order; CS: Covid-19 sample; RS: reference sample (see Supp. File 7)

### The reasons for UNC

Among the studies using the MISSCARE Survey, four [39, 45, 49, 55] did not report the reasons item by item. In the remaining, “Inadequate number of staff” (e.g., in Wave 1 and Wave 2 sample of Falk et al. [38]; [25]) was reported as the most significant reason in six studies, “Unexpected raise in patient volume and/or acuity” as the first or second reason in four studies (e.g., [38, 48]), and “Urgent patient situations” as the first, second, or third in six studies (e.g., [41, 47]) (Table 2, Supplementary Table 3). The reasons for UNC that were given least were “Other departments did not provide the needed care”, “Inadequate hand-off from previous shift or sending unit”, “Caregiver is off unit or unavailable”, and “Tension or communication breakdowns with the medical staff/other support departments” (Table 2, Supplementary Table 3).

Regarding the findings from the UNCS [37], “Priority setting” and “Supervision of nursing aides” were reported as the most frequent factors causing UNC, followed by “Communication”. In particular, the most frequent reasons were “Inaccurate initial priority setting”, “Tension/conflicts within the nursing staff”, and “Inadequate nursing care model (e.g., functional task-oriented model of care)”. The reasons given least were the material and human resources as well as the unpredictability of the workflows (Table 6, Supplementary File 7).

In 22 studies, UNC has been linked to other, additional factors. Among these, organisational factors, insufficient resources, and large hospital facilities were reported as increasing UNC [40, 45]; other factors (e.g., adequate staff, the quality of care, the safety of the patients in the unit, a favourable nursing work environment, and the perceived accountability, organisational support, and leadership) hindered the occurrence of UNC (Table 7). Among the work-related factors, the type of shift work (afternoon shift [35]; 12-hour shift [41]; both day and night shift (not only night shift) [47]), overtime work, the type of unit, the workloads, and other factors increased the occurrence of UNC, whereas having a few patients to each nurse or COVID-19 patients, or better staffing levels, all decreased the occurrence of UNC (Table 7). Moreover, at the individual level, less than 10 years of experience and several other factors close to the nurses’ emotional state and well-being all decreased the occurrence of UNC (Table 7).

**Table 7:** The additional unfinished nursing care-related factors emerged in the studies (= 22)

	Albsoul et al. [34]	Alfuqaha et al. [35]	Al Muharraq et al. [36]	Cengia et al. [37]	Falk et al. [38]	Gurkova et al. [25]	Gurkova et al. [39]	Hackman et al. [40]	Hosseini et al. [41]	Jarozs & Młynarska, 2023	Jarozs et al., 2022	Khrais et al. [44]	Labrague et al. [45]	Magsoud et al., 2022	Mingude et al. [47]	Rahmani et al. [49]	Schneider-Matyka et al. [50]	Tomaszewska et al. [51]	Uchmanowicz et al. [52]	Vincelette et al. [53]	Xie et al. [55]	Zhang et al. [22]
**Reasons (considered as increased)**	**Unfinished Nursing Care Occurrence**																					
**Organizational/ institutional**																						
Insufficient resources								↑														
Large hospital facilities													↑									
Adequate staffing on the unit					**-**																	↓
Quality of care on the unit					**-**															↓		
Patient safety on the unit					**-**								↓			↓				↓		
Favourable nursing working environment							↓											↓		↓		↓
Accountability												↓										
Perceived organizational support												↓										
Level of leadership ability																						↓
**Work related**																						
Shift work type		↑							↑						↑							
Overtime work						↑	↑								↑							
Unit types				↑**			↑**															
Hospital types	↑*					↑*																
Workload/role overload														↑							↑	↑
Unexpected situations in work units								↑														
Activities without collaboration with the resident								↑														
Number of patients in the last shift						↑																
The leader–member exchange																					↑	
Negative residents’ characteristics								↑														
Training needs																						↑
Patient to nurse ratio												↓										
Number of COVID-19 patients in the unit												↓										
Staffing levels													↓									
**Individual**																						
Less than 10 years of experience		↑							↑			↑			↑			↑				
Age		↑										↑						↑	↑			
Satisfaction with the financial situation										↑		↑										
Emotional exhaustion														↑					↑			
Number of diseases occurring in the nurses										↑												
Satisfaction with the place of residence										↑												
Intention to leave			↑		**-**																	
Feeling fatigue											↑											
Depersonalization																			↑			
Level of perceived stress																	↑					
Professional burnout																			↑			
Job satisfaction		↓	↓		**-**	↓				↓		↓			↓				↓			
Educational level															↓				↓			
Work addiction																					↓	
Cognitive functions of nurses											↓											
Individual characteristics																						↓
More than 10 years of experience											↓											
Satisfaction with teamwork at the workplace						↓																

↑, significantly increased; ↓, significantly decreased; -, not significant; B, before pandemic; D, during pandemic

* According to Albsoul et al. [34] there were statistically significant variations between the hospital sectors in terms of labour resources, material resources and communication. However, Gurkova et al. [39] found no distinctions between the hospital and UNC sectors’ explanations. Public hospitals had more “material resources” than private hospitals, while university hospitals had more “communication issues” than either public or private hospitals [34]. Private hospitals had more “labour resources”” than public and university hospitals. According to Gurkova et al. [39], there were notable variations between UNC’s people and material resources and those of the hospital sector, but there were no noteworthy differences in UNC’s communication component

** While Gurkova et al. [25] did not distinguish between surgical and internal medicine wards, Cengia et al. [37] found a slightly different significance on priority setting and human resources issues, which were perceived at higher significance among nurses working in COVID-19 units compared with non-COVID-19 units

### The Main consequences of UNC

No studies reported the consequences of UNC.

## Discussion

At the overall level, a total of 25 studies conducted mainly in European and Asiatic countries were produced during the pandemic, around 10 studies a year, continuing the tradition of this research field during difficult times for both nurses and healthcare settings. All tools available in the field were used, mostly the MISSCARE Survey, but also, on fewer occasions, BERNCA, also in its revised forms. As previously, mostly cross-sectional studies along with a few comparative studies were produced, suggesting the likelihood of a merely descriptive intent due to the challenging times. The order of UNC interventions that emerged across studies is substantially in line with pre-pandemic data, while some interesting variations emerged at the country and inter-country levels. Labour resources and reasons close to the emotional state and well-being of nurses were mentioned as most affecting UNC during the pandemic. However, none of the studies investigated the consequences of the phenomenon.

The discussion section follows the results structure and includes a reflection on the methodological quality of the studies and UNC occurrence, reasons, and consequences.

### Included studies and their methodological quality

Studies released after the World Health Organisation declared the COVID-19 pandemic [30] as a period characterised by altered working conditions, workloads, and processes compared to those of the pre-pandemic era were included. No UNC differences between COVID-19 and non-COVID-19 patients emerged [63, 64], suggesting that the pandemic affected the whole system. Moreover, given the substantial disruption of the routine care processes in the health systems, which may require time to recover, and with the likelihood of not reaching the same levels of the pre-pandemic era, a comprehensive review may contribute to providing a new reference point for future studies in the field of UNC.

Fewer than 10 studies a year were produced, in line with the pre-pandemic era [64, 65]; moreover, data collection was performed mainly in 2020 and 2021, suggesting that available findings reflect the first phases of the pandemic. The leading continents in these studies were Europe and Asia, unlike in the past when the United States was the leading country, given that the missed care/left undone concepts were developed there [2]. Asian and European countries were those firstly and dramatically hit by the pandemic, thus triggering researchers to measure the UNC. However, the setting of the data collection has remained the hospital, as in the pre-pandemic era [66]: this finding is in line with the expanded capacity required in the hospitals and the recognition of their key role, especially in some waves, in facing the pandemic. Interestingly, several studies involved more units in very different institutions (e.g., [47]), which seems to suggest that this research line was scaled up during the pandemic from unit-based studies to large healthcare systems, thus embodying a reasonable health service research perspective because the whole system was changed to provide the care, and no one single part was left unaltered.

The study designs were cross-sectional with some comparative examples, as documented in the pre-pandemic era (e.g., [29]). The turbulent environments may have prevented longitudinal studies (e.g., to discover UNC outcomes). Forty-three [48] to 2,700 [40] nurses, nursing assistants, and care workers were involved, the sample sizes mirroring those of the pre-pandemic era [66]. However, no studies involved midwives, which suggests a lack of evidence in terms of what happened in maternal and paediatric departments.

Four different tools have been used to measure UNC, from those most validated across the world, namely the MISSCARE Survey [39] to more recent instruments, such as the ICU-ONC [53]. The different instruments used reflect the trends in this research field, characterised by a range of validated tools, thus preventing comparisons across studies. On the one hand, the utilisation of classic, well-validated tools may have provided accurate data and increased the comparison with pre- and intra-pandemic studies, whereas on the other hand, tools designed for a non-pandemic situation may have failed in their capacity to detect UNC in extraordinary conditions. Moreover, all tools collected UNC data as perceived by nurses, and their perceptions may have been influenced by the stress and the dramatic working conditions they were experiencing, as well as by the desire to do the best for the patients.

The overall quality of the studies was methodologically good: the extraordinary difficulties posed by the pandemic also required new strategies (e.g., to promote study participation among nurses, design protocols, and initiate studies while other priorities are perceived) in conducting research and seem to have been faced appropriately by researchers.

### The occurrence of UNC

The different UNC activities, in their order, can be discussed around three main perspectives: (1) the instrument used; (2) the intercountry and intra-countries differences; and (3) the state of the evidence in the pre-pandemic era. The order of UNC interventions emerged across studies, for some countries are substantially in line with pre-pandemic data. The MISSCARE Survey studies highlighted that, during the pandemic, nurses firstly postponed or omitted interventions that call for proximity to the patient, such as oral care, or one-on-one interaction, such as ambulation. Studies using the ICU-ONC tool also showed the same trend, suggesting that these two tools can detect actions of care at the bedside. Nursing interventions related to organisation and communication were instead commonly unfinished in studies using the PIRNCA scale. Communication should also be seen as a fundamental care [67–69], as speaking and listening were most often seen as a nursing necessity during the pandemic. Differently, education, disinfection measures, and monitoring were the most frequent UNC activities in studies employing the BERNCA scale. Likewise, nursing interventions for patient follow-up were frequently unfinished in a study using the UNCS [37].

The most significant nursing interventions identified during the pandemic were monitoring, educating the patient, and implementing preventive measures against infections. Nurses may have felt that their usual applications were inadequate or incomplete given the growing demand for these interventions, or they may have believed that they would be unable to complete these applications out of fear of failing. Finally, social and rehabilitative nursing interventions were ranked first as unfinished activities in studies using the BERNCA-NH instrument. This reflects the contingencies of the COVID-19 pandemic, which forced residents of nursing homes to remain in their own rooms [70]. Therefore, at the overall level, it seems that nurses adopted the pre-pandemic patterns of prioritisation (e.g., failing in ensuring fundamental care) with the intent of reducing exposure in patients’ rooms for an extended period and to avoid the source of contagion [71], and/or due to the fatigue caused by the personal protective equipment worn (e.g., [72]). The rationed nursing activities did not turn out to be very different from those of the pre-pandemic period (e.g., [2, 73]), as also emerged in those studies that included comparative studies [35, 38].

However, interesting intra- and inter-country differences have emerged: at the intra-country level, two main patterns are evident. In Sweden, for example, Falk et al. [38] and von Vogelsan et al. [54] found that the three most unfinished activities are substantially the same, whereas in Jordan [35, 44] and Iran [41, 49], the first three unfinished activities differ (Table 2, Supplementary Table 3). Similarly, at the inter-country level, in those studies using the MISSCARE Survey performed across Europe, the unfinished activities seem to have similar trends in the order pattern. Comparing these countries with those where UNC has started to be measured (e.g., Iran, Jordan, Saudia Arabia, Indonesia, Sultanate of Oman), feeding the patient and offering emotional support were not missed immediately, while attending interdisciplinary meetings was unfinished at first. In the two studies using the BERNCA-NH tool, a similar divergence appeared: in the study by Zhang et al. [22] performed in China, some activities (i.e., providing emotional support and rehabilitation care) were the first to be unfinished, while in Hackman et al. [40] these were ranked as being missed less often. Examples can also be found in studies using the PIRNCA and performed in Poland [50] and Indonesia [56]. On the one hand, this seems to suggest that when the healthcare system is under tremendous pressure, as during the pandemic, the process of prioritisation is based on pre-established patterns (e.g., across Europe; [74]); on the other hand, different patterns seem to be enacted outside of Europe, mainly in Asiatic countries. Given that these countries are substantially new to measuring UNC, replicating studies to establish whether the emerged patterns are the same as those used in normal conditions is strongly recommended.

Above all, studies produced during the pandemic period report unfinished activities according to the tool used. For example, the MISSCARE Survey was developed in the early 2000s [59] and is able to measure “basic” nursing activities; therefore, its capacity to detect exactly what happened in the nursing processes during the pandemic should be debated.

### The reasons for UNC

First, issues regarding human resources and the increased needs of patients were the most cited reasons in those studies using the MISSCARE tool, while issues among the staff or across departments impacted only a little. This is likely derived from the expanded capacity of the health systems under urgent circumstances [75] that increased the well-known shortages in resources, whereas facing the pandemic reduced tensions within the staff and across units, promoting a sense of collaboration [76, 77]. Moreover, nurses became infected and were not available when quarantined: all these situations seriously disrupted the capacity of nursing care [21, 22], threatening the patients’ needs [16, 17, 78]. Conversely, for Cengia et al. [37], human resources were not an issue in triggering UNC occurrence; however, this is a single study with the UNC survey tool, and although performed in several facilities, its findings may be interpreted from different perspectives: the units involved in the study may have been better equipped during the pandemic to deal with the situation, or nurses may have learnt for several years how to work under pressure, with limited resources, in a sort of “normalised” condition, where working under such conditions was not an issue [63].

Other potential reasons documented among studies are in line with those documented by Chiappinotto et al. [29]. However, two new elements emerged at the overall level among studies performed during the pandemic. Firstly, in those cases where the same reason has been documented (e.g., the role of working overtime [25, 39, 47]), no conflicting findings have been reported across studies, suggesting an evident accumulation of knowledge in the same direction. Previously, conflicting findings emerged for the same reasons across studies, in some increasing and in others hindering the occurrence of UNC (e.g., working overtime [29]). The increased homogeneity of the findings that emerged in the pandemic studies may depend on the same circumstances experienced in all healthcare services across the world. Secondly, several emotional factors at the nurses’ level (e.g., satisfaction, burnout, satisfaction with economic situation, stress) have been investigated and associated with UNC. The focus seems to be the professional and personal well-being of the nurses, reasons that may have a role as antecedents of UNC but that also express the consequences of the unfinished care phenomenon itself as well as the consequences of the exacerbated working conditions during the pandemic.

### The Main consequences of UNC

No UNC consequences have been documented to date confirming the tradition of this research field in which outcomes are under-reported [79]. In difficult times with turbulent environments, unstable staff, and disconnections between healthcare settings (e.g., hospital and community settings), it would be difficult to link the occurrence of UNC to the different potential outcomes at the patient, nurse, and organisational levels [5, 12–14]. However, the occurrence of UNC may have bolstered the negative effects of other widely observed phenomena, such as the decreased accessibility and continuity of care observed during the pandemic, thus indirectly affecting the health outcomes at both the individual and collective levels (e.g., reduced screening, reduced care for cancer patients) [80, 81].

### Limitations

This review has several limitations. First, databases were searched using well-known established keywords in the field, strictly connected with the conceptual definitions in the field and with the tools measuring the phenomenon. Moreover, given that no MeSH terms have been established in the field, researchers used keywords. Consequently, some studies may have been missed. Second, studies whose data collection period was uncertain or ambiguous (e.g., started before or during the pandemic) were excluded. Moreover, studies not using validated instruments with available reliability and validity data were also excluded, and these decisions may have introduced a selection bias. Furthermore, grey literature was not assessed, introducing additional selection bias. Third, we included only articles written in English, Turkish, or Italian, so the comprehensiveness of this review could have been threatened by the exclusion of other languages. Fourth, in the data analysis and synthesis process, an approach was adopted aiming at ensuring accuracy given the different measurement tools used in the field. Moreover, the data analysis process was conducted in an innovative manner by considering each intervention or reason at the granular level (the order, according to the statistical values) instead of the global level (global scores). This may have provided clarity, but it may have compromised the depiction of a global picture of the phenomenon. No previous similar approaches have been used in this field. Accumulating evidence with additional studies, such as summarising findings in the post-pandemic era, may corroborate the analytical strategy used.

## Conclusion

UNC studies produced during the pandemic documented the occurrence of the phenomenon and its reasons mainly in the first and second waves of the COVID-19 pandemic. These studies were conducted mainly in Europe and Asia, which were the first to be dramatically affected by the pandemic. The studies involved multicentre units in the attempt to measure the whole response of the healthcare settings, mainly using the MISSCARE Survey with descriptive intents and using quality, sound research methodologies.

At the overall level, those nursing care activities that were mostly unfinished during the pandemic are substantially the same as those reported in the pre-pandemic era, suggesting that nurses applied the same prioritisation responses in difficult times. However, interesting intra- and inter-country differences emerged: those countries new to measuring unfinished care reported different patterns compared to those seen in Europe and the US, where this research is well established; they also reported intra-country variations, suggesting an interesting new course of research in the field. The new patterns that emerged should be better investigated through post-pandemic studies to discover whether they reflected the decision-making process during difficult conditions or a different prioritisation process.

Across studies, the primary reasons for UNC were listed as labour resources, followed by other specific reasons related to organisational, work, and individual variables. Substantially, the evidence is in line with that previously documented. However, findings are consistent across studies, suggesting that health services experienced similar pressure worldwide. Moreover, several emotional factors have been investigated among nurses, revealing their important role in triggering UNC. This level should be investigated further, considering the long-term consequences of the pandemic on the well-being of the workforce. Given that no studies have attempted to measure the UNC consequences, more efforts are also required in this direction.

### Electronic supplementary material

Below is the link to the electronic supplementary material.

Supplementary Material 1

Supplementary Material 2

Supplementary Material 3

Supplementary Material 4

Supplementary Material 5

Supplementary Material 6

Supplementary Material 7

Supplementary Material 8

## Data Availability

All data generated or analysed during this study are included in this published article [and its supplementary information files].

## Abbreviations

BERNCA: Basel Extend of Rationing of Nursing Care
BERNCA-NH: Basel Extent of Rationing of Nursing Care for Nursing Homes
BERNCA-R: Basel Extent of Rationing of Nursing Care Revised
CINAHL: Cumulative Index to Nursing and Allied Health Literature
COVID-19: Coronavirus-19
ICU-ONC: Intensive Care Unit Omitted Nursing Care instrument
PIRNCA: Perceived Implicit Rationing of Nursing Care
PRISMA: Preferred Reporting Items for Systematic Reviews and Meta Analysis
UNC: Unfinished Nursing Care
UNCS: Unfinished Nursing Care Survey
WHO: World Health Organization
