# Supplementary material for: Unfinished nursing care in healthcare settings during the COVID-19 pandemic: a systematic review
Source: BMC Health Serv Res. 2024 Mar 19;24:352. doi: 10.1186/s12913-024-10708-7 (PMC10949800; doi:10.1186/s12913-024-10708-7)
Supplement: Supplementary file 1 — Supplementary Material 1 [file 12913_2024_10708_MOESM1_ESM.docx]

**Supplementary Table 1.** Keywords and search strings of searching databases

| **PUBMED** | | | |  |
| --- | --- | --- | --- | --- |
| **Query** | **Filters** | **Search Details** | **Results** | **Time** |
| 1 | Time: From March 2020 to May 2023 | ("nurse"[Title/Abstract] OR "nursing"[Title/Abstract]) AND ("missed care"[Title/Abstract] OR "missed nursing care"[Title/Abstract] OR "unfinished nursing care"[Title/Abstract] OR "unfinished care"[Title/Abstract] OR "implicit rationing of nursing care"[Title/Abstract] OR "implicit rationing"[Title/Abstract] OR "rationing of nursing care"[Title/Abstract] OR "rationed care"[Title/Abstract] OR "prioritization process"[Title/Abstract] OR "omitted nursing care"[Title/Abstract] OR "task left undone"[Title/Abstract] OR "task undone"[Title/Abstract]) | 298 | 05/05/2023 |
| **CINAHL** | | | | |
| **Query** | **Filters** | **Search Details** | **Results** | **Time** |
| 1 | Time: From March 2020 to May 2023: | TI ( nurse OR nursing ) OR AB ( nurse OR nursing ) AND TI ( “missed care” OR “missed nursing care” OR "unfinished nursing care" OR "unfinished care" OR "implicit rationing of nursing care" OR "implicit rationing" OR "rationing of nursing care" OR "rationed care" OR "prioritization process" OR "omitted nursing care" OR "task left undone" OR "task undone" ) OR AB ( “missed care” OR “missed nursing care” OR "unfinished nursing care" OR "unfinished care" OR "implicit rationing of nursing care" OR "implicit rationing" OR "rationing of nursing care" OR "rationed care" OR "prioritization process" OR "omitted nursing care" OR "task left undone" OR "task undone" ) | 186 | 05/05/2023 |
| **SCOPUS** | | | | |
| **Query** | **Filters** | **Search Details** | **Results** | **Time** |
| 1 | Time: From March 2020 to May 2023 | TITLE-ABS-KEY(nurse OR nursing) AND TITLE-ABS-KEY("missed care" OR "missed nursing care" OR "unfinished nursing care" OR "unfinished care" OR "implicit rationing of nursing care" OR "implicit rationing" OR "rationing of nursing care" OR "rationed care" OR "prioritization process" OR "omitted nursing care" OR "task left undone" OR "task undone" ) OR ( "missed care" OR "missed nursing care" OR "unfinished nursing care" OR "unfinished care" OR "implicit rationing of nursing care" OR "implicit rationing" OR "rationing of nursing care" OR "rationed care" OR "prioritization process" OR "omitted nursing care" OR "task left undone" OR "task undone") | 905 | 05/05/2023 |

**Legend:** CINAHL, Cumulative Index to Nursing and Allied Health Literature.
