# Supplementary material for: Unfinished nursing care in healthcare settings during the COVID-19 pandemic: a systematic review
Source: BMC Health Serv Res. 2024 Mar 19;24:352. doi: 10.1186/s12913-024-10708-7 (PMC10949800; doi:10.1186/s12913-024-10708-7)
Supplement: Supplementary file 2 — Supplementary Material 2 [file 12913_2024_10708_MOESM2_ESM.docx]

**Supplementary Table 2.** Quality appraisal of included studies assessed with Joanna Briggs Institute tool for analytical cross-sectional studies [32]

| **Included Studies** | **Item 1**. Were the criteria for inclusion in the sample clearly defined? | | | **Item 2**. Were the study subjects and the setting described in detail? | | | **Item 3**. Was the exposure measured in a valid and reliable way? | | | **Item 4**. Were objective, standard criteria used for measurement of the condition? | | | **Item 5**. Were confounding factors identified? | | | **Item 6**. Were strategies to deal with confounding factors stated? | | | **Item 7**. Were the outcomes measured in a valid and reliable way? | | | **Item 8**. Was appropriate statistical analysis used? | | |
| --- | --- | --- | --- | --- | --- | --- | --- | --- | --- | --- | --- | --- | --- | --- | --- | --- | --- | --- | --- | --- | --- | --- | --- | --- |
|  | **R^1^** | **R^2^** | **C** | **R^1^** | **R^2^** | **C** | **R^1^** | **R^2^** | **C** | **R^1^** | **R^2^** | **C** | **R^1^** | **R^2^** | **C** | **R^1^** | **R^2^** | **C** | **R^1^** | **R^2^** | **C** | **R^1^** | **R^2^** | **C** |
| Albsoul et al. [43] | Y | Y | **Y** | Y | Y | **Y** | Y | Y | **Y** | NA | NA | **NA** | N | Y | **Y** | N | Y | **N** | Y | Y | **Y** | Y | Y | **Y** |
| Alfuqaha et al. [38] | Y | Y | **Y** | Y | Y | **Y** | Y | Y | **Y** | NA | Y | **NA** | Y | Y-U | **Y** | Y | Y-U | **U** | Y | Y | **Y** | Y | Y | **Y** |
| Al Muharraq et al. [59] | Y | Y | **Y** | Y | Y | **Y** | Y | Y | **Y** | NA | NA | **NA** | Y | Y | **Y** | U | Y-U | **Y** | Y | Y | **Y** | Y | Y | **Y** |
| Cengia et al. [45] | U | Y | **Y** | Y | Y | **Y** | Y | Y | **Y** | NA | Y | **NA** | N | N | **N** | N | N | **N** | Y | Y | **Y** | Y | Y | **Y** |
| Falk et al. [44] | U | Y | **Y** | Y | Y | **Y** | Y | Y | **Y** | NA | Y | **NA** | N | Y-U | **Y** | N | Y-U | **U** | U | Y | **Y** | Y | Y | **Y** |
| Gurková et al. [58] | Y | Y | **Y** | Y | Y | **Y** | Y | Y | **Y** | NA | NA | **NA** | Y | Y | **Y** | U | Y | **Y** | Y | Y | **Y** | Y | Y | **Y** |
| Gurková et al. [57] | Y | Y | **Y** | Y | Y | **Y** | Y | Y | **Y** | NA | NA | **NA** | Y | Y | **Y** | Y | N | **N** | Y | Y | **Y** | Y | Y | **Y** |
| Hackman et al. [52] | Y | Y | **Y** | Y | Y | **Y** | Y | Y | **Y** | NA | NA | **NA** | Y | Y | **Y** | U | Y | **U** | Y | Y | **Y** | Y | Y | **Y** |
| Hosseini et al. [51] | Y | Y | **Y** | U | N | **U** | Y | Y | **Y** | NA | NA | **NA** | Y | Y | **Y** | U | Y | **U** | Y | Y | **Y** | Y | Y | **Y** |
| Jarosz et al. [53] | Y | Y | **Y** | U | Y | **U** | Y | Y | **Y** | NA | NA | **NA** | Y | Y | **Y** | Y | U | **Y** | Y | Y | **Y** | Y | Y | **Y** |
| Jarosz & Mlynarska [34] | Y | Y | **Y** | U | U | **U** | Y | Y | **Y** | NA | NA | **NA** | Y | Y | **Y** | Y | Y | **Y** | Y | Y | **Y** | Y | Y | **Y** |
| Khrais et al. [55] | Y | Y | **Y** | Y | Y | **Y** | Y | Y | **Y** | NA | NA | **NA** | Y | Y | **Y** | Y | U | **Y** | Y | Y | **Y** | Y | Y | **Y** |
| Labrague et al. [46] | Y | Y-U | **Y** | Y | Y | **Y** | Y | Y | **Y** | NA | NA | **NA** | Y | Y | **Y** | Y | Y | **Y** | Y | Y | **Y** | Y | Y | **Y** |
| Maghsoud et al. [56] | Y | Y | **Y** | U | N | **U** | Y | Y | **Y** | NA | NA | **NA** | Y | Y | **Y** | Y | Y | **Y** | Y | Y | **Y** | Y | Y | **Y** |
| Mingude et al. [47] | Y | Y | **Y** | Y | Y | **Y** | Y | Y | **Y** | NA | NA | **NA** | Y | Y | **Y** | Y | U | **Y** | Y | Y | **Y** | Y | Y | **Y** |
| Nymark et al. [42] | N | N-U | **U** | Y | Y | **Y** | Y | Y | **Y** | NA | N-Y | **NA** | N | Y-U | **Y** | N | Y-U | **U** | Y | Y | **Y** | Y | Y | **Y** |
| Rahmani et al. [60] | Y | Y | **Y** | Y | Y | **Y** | Y | Y | **Y** | NA | NA | **NA** | Y | Y | **Y** | U | Y | **Y** | Y | Y | **Y** | Y | Y | **Y** |
| Schneider-Matyka et al. [35] | Y | Y | **Y** | U | N-U | **U** | Y | Y | **Y** | NA | NA | **NA** | Y | Y | **Y** | U | Y | **Y** | Y | Y | **Y** | Y | Y | **Y** |
| Tomaszewska et al. [50] | Y | Y | **Y** | U | N | **U** | Y | Y | **Y** | NA | NA | **NA** | Y | Y | **Y** | U | Y | **U** | Y | Y | **Y** | Y | Y | **Y** |
| Uchmanowicz et al. [54] | Y | Y | **Y** | U | U | **U** | Y | Y | **Y** | NA | NA | **NA** | Y | Y | **Y** | Y | Y | **Y** | Y | Y | **Y** | Y | Y | **Y** |
| Vincelette et al. [48] | Y | Y | **Y** | Y | Y | **Y** | Y | Y | **Y** | NA | NA | **NA** | Y | Y | **Y** | Y | Y | **Y** | Y | Y | **Y** | Y | Y | **Y** |
| von Vogelsan et al. [41] | U | Y | **Y** | Y | Y | **Y** | Y | Y | **Y** | NA | Y | **NA** | Y | N | **Y** | Y | N | **U** | Y | Y | **Y** | Y | Y | **Y** |
| Xie et al. [49] | Y | Y | **Y** | U | Y | **Y** | Y | Y | **Y** | NA | NA | **NA** | Y | Y | **Y** | Y | Y | **Y** | Y | Y | **Y** | Y | Y | **Y** |
| Yuwanto et al. [40] | Y | Y | **Y** | U | Y | **U** | Y | Y | **Y** | NA | NA | **NA** | N | Y | **Y** | N | U | **N** | Y | Y | **Y** | Y | Y | **Y** |
| Zhang et al. [22] | Y | Y | **Y** | Y | Y | **Y** | Y | Y | **Y** | NA | NA | **NA** | Y | Y | **Y** | Y | Y | **Y** | Y | Y | **Y** | Y | Y | **Y** |

**Legend:** Y: yes; N: no; U: unclear; NA: not applicable; C: Consensus; R^1^: Rater 1 (see Authors); R^2^: Rater 2 (see Authors).
