# Supplementary material for: Unfinished nursing care in healthcare settings during the COVID-19 pandemic: a systematic review
Source: BMC Health Serv Res. 2024 Mar 19;24:352. doi: 10.1186/s12913-024-10708-7 (PMC10949800; doi:10.1186/s12913-024-10708-7)
Supplement: Supplementary file 3 — Supplementary Material 3 [file 12913_2024_10708_MOESM3_ESM.docx]

**Supplementary Table 3.** The UNC occurence order and reasons in studies based on MISSCARE Survey (=14) [37]

|  | |  | Order * | | | | | | | | | | | | | | | | | | | | | | |  |  |
| --- | --- | --- | --- | --- | --- | --- | --- | --- | --- | --- | --- | --- | --- | --- | --- | --- | --- | --- | --- | --- | --- | --- | --- | --- | --- | --- | --- |
| PART A, Interventions | | **Albsoul et al. [43]** | **Alfuqaha et al. [38]** | | | **Al Muharraq et al. [59]** | **Falk et al. [44]** | | | | | **Gurkova et al. [58]** | **Gurková et al. [57]** | **Hosseini et al. [51]** | | **Khrais et al. [55]** | **Labrague et al. [46]c** | **Mingude et al. [47]** | **Nymark et al. [42]** | | | | **Rahmani et al. [60]d** | **von Vogelsan et al. [41]** | | **Xie et al. [49]e** |  |
|  | |  | **B** | **D** | |  | **B** | **W^2^** | | **W^3^** | |  |  |  | |  |  |  | **CS** | | **RS** | |  | **CS** | **RS** |  |  |
| Ambulation 3 times per day or as ordered | | - | 1 | 1 | | 2 | 1 | 4 | | 5 | | - | 1 | - | | 4 | 4 | 14 | 3 | | 4 | | 8 | 3 | 3 | - |  |
| Assess effectiveness of medications | | - | 9 | 10 | | 19 | 21 | 15 | | 15 | | - | 8 | - | | 20 | 14 | 11 | 12 | | 16 | | 9 | 4 | 9 | - |  |
| Turning patient every 2 hours | | - | 3 | 2 | | 3 | 4 | 3 | | 2 | | - | 4 | - | | 15 | - | 3 | 1 | | 2 | | 5 | 2 | 1 | - |  |
| Mouth care | | - | 2 | 4 | | 6 | 11 | 17 | | 8 | | - | 7 | - | | 9 | 5 | - | 4 | | 3 | | 3 | 6 | 4 | - |  |
| Patient teaching about procedures, tests, and other diagnostic studies | | - | 4 | 5 | | 5 | 7 | 8 | | 10 | | - | 5 | 1 | | 6 | 13 | 12 | 13 | | 18 | | 7 | 11 | 16 | - |  |
| PRN medication requests acted on within 15 min | | - | 16 | 21 | | 14 | 25 | 15 | | 15 | | - | 12 | - | | 12 | - | 2 | 8 | | 15 | | 10 | 13 | 18 | - |  |
| Full documentation of all necessary data | | - | 19 | 14 | | 13 | 15 | 19 | | 12 | | - | 6 | 5 | | 2 | 12 | 8 | 19 | | 21 | | 10 | 20 | 21 | - |  |
| Feeding patient when the food is still warm | | - | 14 | 6 | | 4 | 3 | 2 | | 1 | | - | 12 | 3 | | 23 | - | - | 10 | | 5 | | 4 | 12 | 8 | - |  |
| Medications administered within 30 min before or after scheduled time | | - | 12 | 16 | | 18 | 10 | 11 | | 9 | | - | 10 | - | | 24 | - | 10 | 21 | | 6 | | 10 | 14 | 7 | - |  |
| Assist with toileting needs within 5 min of request | | - | 10 | 9 | | 8 | 6 | 7 | | 7 | | - | 9 | - | | 13 | - | - | 20 | | 17 | | 3 | 15 | 13 | - |  |
| Response to call light is initiated within 5 min | | - | 15 | 13 | | 10 | 8 | 10 | | 13 | | - | 11 | - | | 3 | - | - | 15 | | 22 | | 10 | 19 | 22 | - |  |
| Emotional support to patient and/or family | | - | 5 | 3 | | 7 | 17 | 9 | | 11 | | - | 3 | 2 | | 14 | 2 | 12 | 11 | | 8 | | 8 | 8 | 6 | - |  |
| Patient bathing/skin care | | - | 8 | 12 | | 9 | 20 | 15 | | 12 | | - | 14 | - | | 19 | - | - | 9 | | 12 | | 3 | 17 | 14 | - |  |
| IV/central line site care and assessments according to hospital policy | | - | 22 | 20 | | - | 12 | 13 | | 12 | | - | 17 | - | | 11 | - | 4 | 7 | | 13 | | 9 | 7 | 10 | - |  |
| Teach patient about plans for their care after discharge | | - | 11 | 15 | | 12 | 2 | 5 | | 4 | | - | 15 | - | | 5 | 8 | 11 | 5 | | 7 | | 10 | 5 | 5 | - |  |
| Monitoring intake/output | | - | 23 | 22 | | 17 | 23 | 20 | | 17 | | - | 16 | 6 | | 18 | 1 | 2 | 18 | | 20 | | 9 | 10 | 15 | - |  |
| Setting up meals for patient who feed themselves | | - | 7 | 11 | | 11 | 3 | 1 | | 3 | | - | 18 | - | | 16 | - | - | 23 | | 9 | | 2 | 22 | 11 | - |  |
| Vital signs assessed as ordered | | - | 21 | 23 | | 22 | 13 | 20 | | 18 | | - | 20 | - | | 17 | - | 6 | 24 | | 24 | | 9 | 24 | 23 | - |  |
| Focused reassessments according to patient condition | | - | 13 | 7 | | 15 | 18 | 12 | | 15 | | - | 13 | - | | 21 | 6 | 7 | 17 | | 14 | | 6 | 18 | 17 | - |  |
| Hand washing | | - | 20 | 24 | | 21 | 16 | 18 | | 14 | | - | 12 | 4 | | 7 | - | - | 16 | | 19 | | 8 | 21 | 20 | - |  |
| Bedside glucose monitoring as ordered | | - | 24 | 18 | | 23 | 22 | 16 | | 16 | | - | 22 | - | | 8 | - | 7 | 22 | | 23 | | 10 | 23 | 24 | - |  |
| Patient assessments performed each shift | | - | 18 | 19 | | 20 | 24 | 19 | | 18 | | - | 21 | - | | 10 | 11 | 12 | 14 | | 10 | | 7 | 16 | 19 | - |  |
| r-Attend interdisciplinary care conferences whenever held | | - | 6 | 8 | | 1 | 5 | 6 | | 6 | | - | - | - | | 1 | - | 5 | 2 | | 1 | | 1 | 1 | 2 | - |  |
| r- Skin/wound care | | - | 17 | 17 | | 16 | 9 | 14 | | 8 | | - | 19 | - | | 22 | 3 | 11 | 6 | | 11 | | 10 | 9 | 12 | - |  |
| r-Interdisciplinary care conferences whenever held | | - | - | - | | - | - | - | | - | | - | 2 | - | | - | 7 | - | - | | - | | - | - | - | - |  |
| r-Discussing about patient expectation | | - | - | - | | - | - | - | | - | | - | - | - | | - | - | 1 | - | | - | | - | - | - | - |  |
| r-pain assesment and management | | - | - | - | | - | - | - | | - | | - | - | - | | - | 10 | 9 | - | | - | | - | - | - | - |  |
| r-Physical examination | | - | - | - | | - | - | - | | - | | - | - | - | | - | - | 11 | - | | - | | - | - | - | - |  |
| r-Initial assesment | | - | - | - | | - | - | - | | - | | - | - | - | | - | - | 13 | - | | - | | - | - | - | - |  |
| r-Review of collected lab result | | - | - | - | | - | - | - | | - | | - | - | - | | - | - | 15 | - | | - | | - | - | - | - |  |
| Treatments and procedures | |  |  |  | |  |  |  | |  | |  |  |  | |  | 9 |  |  | |  | |  |  |  |  |  |
| PART B, Reasons | |  |  |  | |  |  |  | |  | |  |  |  | |  |  |  |  | |  | |  |  |  |  |  |
| Communication | Tension or communication breakdowns within the nursing team | 14 | 15 | | 14 | 14 | 9 | | 8 | | 9 | 2 | - | | - | 2 | - | 12 | | 8 | | 8 | - | 9 | 9 | - | |
|  | Lack of back up support from team members | 12 | 13 | | 12 | 9 | 7 | | 7 | | 7 | 6 | - | | - | 1 | - | 12 | | 6 | | 7 | - | 6 | 7 | - | |
|  | Nursing assistant did not communicate that care was not done | 16 | 16 | | 17 | 17 | 12 | | 8 | | 8 | 7 | - | | 5 | 9 | - | 7 | | 7 | | 9 | - | 7 | 6 | - | |
|  | Care giver is off unit or unavailable | 17 | 17 | | 15 | 15 | 8 | | 6 | | 6 | 6 | - | | - | 13 | - | 11 | | 11 | | 10 | - | 15 | 12 | - | |
|  | Tension or communication breakdowns with the medical staff | 15 | 12 | | 11 | 12 | 12 | | 9 | | 8 | 2 | - | | - | 4 | - | - | | 9 | | 6 | - | 17 | 8 | - | |
|  | Tension or communication breakdowns with other support departments | 13 | 9 | | 10 | 11 | 10 | | 8 | | 11 | 7 | - | | - | 6 | - | 10 | | 13 | | 11 | - | 11 | 11 | - | |
|  | Other departments did not provide the care needed | 11 | 10 | | 16 | 10 | 13 | | 10 | | 9 | 5 | - | | - | 13 | - | 13 | | 14 | | 14 | - | 16 | 13 | - | |
|  | Inadequate hand-off from previous shift or sending unit | 10 | 14 | | 5 | 16 | 14 | | 10 | | 10 | 6 | - | | - | 8 | - | 14 | | 10 | | 16 | - | 12 | 15 | - | |
|  | Unbalanced patient assignments | 20 | 7 | | 13 | 6 | 4 | | 4 | | 4 | 4 | - | | 3 | 3 | - | 10 | | 4 | | 5 | - | 4 | 5 | - | |
|  | r-Inadequate supervision of nursing assistants | 18 | - | | - | - | - | | - | | - | - | - | | - | - | - | - | | - | | - | - | - | - | - | |
|  | r-Lack of cues-reminders | 19 | - | | - | - | - | | - | | - | - | - | | - | - | - | - | | - | | - | - | - | - | - | |
| Material resources | Supplies-equipment not available when needed | 8 | 8 | | 4 | 8 | 11 | | 12 | | 12 | 2 | - | | 4 | 13 | - | 2 | | 12 | | 13 | - | 10 | 16 | - | |
|  | Supplies-equipment not function properly when needed | 9 | 6 | | 8 | 5 | 15 | | 11 | | 12 | 5 | - | | 6 | 12 | - | 4 | | 17 | | 17 | - | 13 | 17 | - | |
|  | Medications were not available when needed | 7 | 11 | | 2 | 13 | 6 | | 8 | | 10 | 3 | - | | - | 9 | - | 5 | | 16 | | 12 | - | 8 | 10 | - | |
| Labor resources | Unexpected rise in patient volume and-or acuity on the unit | 2 | 4 | | 6 | 3 | 2 | | 2 | | 1 | 4 | - | | - | 11 | - | 6 | | 1 | | 1 | - | 1 | 1 | - | |
|  | Urgent patient situations | - | 5 | | 3 | 7 | 3 | | 3 | | 3 | 6 | - | | 2 | 5 | - | 1 | | 2 | | 2 | - | 2 | 2 | - | |
|  | Inadequate number of staff | 1 | 1 | | 1 | 1 | 1 | | 1 | | 2 | 1 | - | | 1 | 10 | - | 3 | | 3 | | 3 | - | 3 | 3 | - | |
|  | Inadequate number of assistive personnel | 3 | 3 | | 9 | 2 | 9 | | 5 | | 7 | 2 | - | | - | 7 | - | 9 | | 15 | | 15 | - | 14 | 14 | - | |
|  | r-Emotional or physical exhaustion | 4 | - | | - | - | - | | - | | - | - | - | | - | - | - | - | | - | | - | - | - | - | - | |
|  | r-Interruptions-Multitasking | 5 | - | | - | - | - | | - | | - | - | - | | - | - | - | - | | - | | - | - | - | - | - | |
|  | r-Inadequate support from leadership | 6 | - | | - | - | - | | - | | - | - | - | | - | - | - | - | | - | | - | - | - | - | - | |
|  | r-Heavy admission and discharge activity | - | 2 | | 7 | 4 | 5 | | 5 | | 5 | 3 | - | | - | 11 | - | 8 | | 5 | | 4 | - | 5 | 4 | - | |

**Legend:** -, not described; r-, revised item; B, before pandemic; D, during pandemic; W, Wave of pandemic; CS, Covid-19 Sample; RS, Reference Sample

^a^, this study was concentrated on analyzing only the elements in Part B in the MISSCARE Survey; ^b^, this study defined the part B of MISSCARE Survey; ^c^, this study defined a few specific items of part A within the main score of MISSCARE Survey; ^d^, this study defined only the results of part A of MISSCARE survey and overall score of survey; ^e^, this study defined only the overall score of MISSCARE survey; *the order (e.g., 1,2,3) were determined according to the statistical values reported in the articles.
