# Supplementary material for: Unfinished nursing care in healthcare settings during the COVID-19 pandemic: a systematic review
Source: BMC Health Serv Res. 2024 Mar 19;24:352. doi: 10.1186/s12913-024-10708-7 (PMC10949800; doi:10.1186/s12913-024-10708-7)
Supplement: Supplementary file 4 — Supplementary Material 4 [file 12913_2024_10708_MOESM4_ESM.docx]

**Supplementary Table 4.** The UNC occurence in studies based on the Perceived Implicit Rationing of Nursing Care- PIRNCA (=4) [1]

| Interventions | Order* | | | |
| --- | --- | --- | --- | --- |
|  | **Jarosz et al. [53]^a^** | **Jarosz et al. [34]^a^** | **Schneider-Matyka et al. [35]** | **Yuwanto et al. [40]** |
| Assistance with physical care |  |  |  |  |
| Routine hygiene | - | - | 14 | 5 |
| Routine skin care | - | - | 17 | 1 |
| Change soiled linen | - | - | 11 | 11 |
| Assist with ambulation | - | - | 8 | 4 |
| Assist with repositioning | - | - | 7 | 10 |
| Assist with bowel and bladder elimination | - | - | 13 | 3 |
| Assist with oral nutrition and hydration | - | - | 20 | 6 |
| Promote physical comfort/pain control | - | - | 16 | 12 |
| Implementation of prescribed treatment plan | - | - |  |  |
| Medication administration | - | - | 29 | 20 |
| Enteral and parenteral nutrition | - | - | 30 | 14 |
| Wound care | - | - | 27 | 15 |
| Dressing changes | - | - | 28 | 18 |
| IV therapy | - | - | 22 | 9 |
| Infection control practices | - | - | 26 | 30 |
| Emotional support and teaching |  |  |  |  |
| Teaching patient or family | - | - | 9 | 13 |
| Prepare patient or family for treatments/procedures | - | - | 25 | 23 |
| Offer emotional or psychological support | - | - | 1 | 8 |
| Surveillance/vigilance |  |  |  |  |
| Monitoring physiological status | - | - | 24 | 17 |
| Monitoring behavior | - | - | 10 | 7 |
| Monitoring safety | - | - | 18 | 21 |
| Follow-up on status changes/requests/unclear orders | - | - | 19 | 22 |
| Timely response to requests | - | - | 4 | 24 |
| Supervise delegated tasks | - | - | 6 | 19 |
| Evaluate the plan of care | - | - | 15 | 27 |
| Coordination of care and discharge planning |  |  |  |  |
| Converse with team members | - | - | 2 | 28 |
| Converse with external agency | - | - | 3 | 2 |
| Converse with patient regarding discharge | - | - | 5 | 29 |
| Documentation |  |  |  |  |
| Review documentation | - | - | 6 | 16 |
| Document initiation/revision of plan of care | - | - | 12 | 26 |
| Document assessments and monitoring activities | - | - | 23 | 25 |
| Document care/interventions | - | - | 21 | 28 |

^a^, this study focused only the overall score of tool; *the order (e.g., 1,2,3) were determined according to the statistical values reported in the articles.
