# Supplementary material for: Unfinished nursing care in healthcare settings during the COVID-19 pandemic: a systematic review
Source: BMC Health Serv Res. 2024 Mar 19;24:352. doi: 10.1186/s12913-024-10708-7 (PMC10949800; doi:10.1186/s12913-024-10708-7)
Supplement: Supplementary file 5 — Supplementary Material 5 [file 12913_2024_10708_MOESM5_ESM.docx]

**Supplementary Table 5.** The UNC occurance in studies based on the Basel Extend of Rationing of Nursing Care (BERNCA) (=1) [79], BERNCA-R (=2) [79] and Basel Extent of Rationing of Nursing Care for nursing homes tool (BERNCA-NH) (=2) [80]

| BERNCA  Interventions | Order* | BERNCA-R  Interventions | Order* | | BERNCA-NH  Interventions | Order* | |
| --- | --- | --- | --- | --- | --- | --- | --- |
|  | **Maghsoud et al. [56]^a^** |  | **Tomaszewska et al. [50]** | **Uchmanowicz et al. [54]^b^** |  | **Hackman et al. [52]** | **Zhang et al. [22]** |
| 1. Activity of Daily Livings (ADLs) |  | Sponge bath | 10 | - | **Activities of daily living (ADL)/Routine Care** | 3** |  |
| (1a) Bathing/skin care | - | Partial sponge bath | 20 | - | Sponge bath/partial sponge bath/skin care | 16 | 19 |
| (1b) Perform oral or dental hygiene for patients | - | Skin care | 31 | - | Oral hygiene | 8 | 15 |
| (1c) Eating | - | Oral hygiene | 28 | - | Assist dressing/undressing | 20 | 17 |
| (1d) Mobilization/changing positions | - | Dental hygiene | 22 | - | Assist food intake | 17 | 16 |
| (1e) Managing body waste (urine, stool, vomit) | - | Assist food intake | 30 | - | Assist drinking | 19 | 18 |
| (1f) Changing bed linen |  | Mobilization | 27 | - | Mobilization/change of the position | 10 | 14 |
| 2. Caring-Support |  | Change of the position | 14 | - | **Caring, rehabilitation, and monitoring** | 2** |  |
| (2a) Emotional or psychosocial support | - | Change of the bed linen | 32 | - | Leave a resident in urine/stool longer than 30 minutes | 13 | 12 |
| (2b) Conversations with patients or their families | - | Emotional & psychological support | 29 | 4 | Emotional support | 7 | 2 |
| 3. Rehabilitation-Instruction-Education |  | Necessary conversation | 18 | - | Necessary conversation with resident or family | 15 | 6 |
| (3a) Toilet training | - | Information about therapies | 11 | - | Toileting/continence training | 12 | 13 |
| (3b) Activating/rehabilitating care | - | Continence training (diapers) | 23 | - | Activating or rehabilitating care | 9 | 1 |
| (3c) Education of patients/their families about self-care | - | Continence training (insert catheter) | 17 | - | Monitoring residents as care workers felt necessary | 14 | 11 |
| (3d) Preparation for hospital discharge | - | Activating or rehabilitating care | 13 | 1 | Monitoring of confuse/cognitively impaired residents & use of restraints/sedatives | 18 | 9 |
| 4. Monitoring-Safety |  | Education and training | 1 | - | Keep residents waiting who rung | 4 |  |
| (4a) Adequate monitoring of patients vital signs | - | Preparation for discharge | 19 | - | **Documentation** |  | 20 |
| (4b) Adequate monitoring of confused/impaired patients | - | Monitoring patients as described by physician | 15 | - | Studying care plans at the beginning of shift | 5 | 8 |
| (4c) Coping with the delayed response of a physician | - | Monitoring patients as the nurse felt necessary | 3 | - | Set up or update residents’ care plans | 6 | 5 |
| (4d) Respond promptly to patient calls | - | Monitoring of confused patients & use of restrains | 24 | - | Documentation of care | 11 | 10 |
| (4e) Adequate hand hygiene | - | Monitoring of confused patients & use of sedatives | 16 | - | **Social care** | 1** |  |
| 5. Documentation |  | Delay in measure because of a physician delay | 6 | - | Scheduled single activity with a resident | 2 | 7 |
| (5a) Review patient documentation at the beginning of the shift | - | Administration of medication, infusions | 21 | 5 | Scheduled group activity with several residents | 3 | 3 |
| (5b) Formulate/update patient care plans | - | Change of wound dressings | 7 | - | Cultural activity for residents with contact outside of nursing home | 1 | 4 |
| (5c) Documentation of performed nursing care | - | Preparation for test and therapies | 5 | - |  |  |  |
|  |  | Keep patient waiting who rung | 25 | - |  |  |  |
|  |  | Adequate hand hygiene | 9 | - |  |  |  |
|  |  | Necessary disinfection measures | 2 | - |  |  |  |
|  |  | Studying care plans | 26 | 2 |  |  |  |
|  |  | Assessment of newly admitted patient | 8 | 3 |  |  |  |
|  |  | Set up care plans | 4 | - |  |  |  |
|  |  | Documentation & evaluation of the care | 12 | - |  |  |  |

* the order (e.g., 1,2,3) were determined according to the statistical values reported in the articles; **, the mean of these main dimensions with the symbol ** have been listed separately from the item scores in the article; a, this study focused only the main score of tool; b, this study defined the first five specific items in the results section within the main score of tool.
