# Supplementary material for: Unfinished nursing care in healthcare settings during the COVID-19 pandemic: a systematic review
Source: BMC Health Serv Res. 2024 Mar 19;24:352. doi: 10.1186/s12913-024-10708-7 (PMC10949800; doi:10.1186/s12913-024-10708-7)
Supplement: Supplementary file 6 — Supplementary Material 6 [file 12913_2024_10708_MOESM6_ESM.docx]

**Supplementary Table 6.** The UNC occurence in the study based on The ICU Omitted Nursing Care instrument (ICU-ONC) (=1) [48]

| Omitted nursing care Order* | Vincelette et al. [48] |
| --- | --- |
| Mobilisation every-two hours | 1 |
| Mouth care for intubated patients | 2 |
| Document treatments and procedures | 3 |
| Timely medication administration | 4 |
| Address new prescriptions, consultations | 5 |
| Treatment and adverse effects surveillance | 6 |
| Venous and arterial catheters care and maintenance | 7 |
| Medication related independent double check | 8 |
| Hemodynamic and physiologic parameters surveillance | 9 |
| Draw labs following prescription | 10 |
| Communicate preoccupations to the medical team | 11 |
| Neurological signs evaluation | 12 |
| Pain assessment (patient unable to communicate) | 13 |
| Provide respiratory care (e.g., aspiration of secretions) | 14 |
| Sedation adjustment based on prescription (e.g., RASS scale) | 15 |
| Respond quickly to alarms indicating potential instability | 16 |
| Pain assessment (patient able to communicate) | 17 |
| Ensure asepsis in treatments or procedures | 18 |
| Intervene rapidly to glucose levels (e.g., IV insulin therapy) | 19 |
| Cardiac monitoring surveillance | 20 |
| Flag the presence of signs or symptoms of infection | 21 |
| Titrate intravenous perfusions for hemodynamic targets | 22 |

*the order (e.g., 1,2,3) were determined according to the statistical values reported in the articles.
