# Supplementary material for: Unfinished nursing care in healthcare settings during the COVID-19 pandemic: a systematic review
Source: BMC Health Serv Res. 2024 Mar 19;24:352. doi: 10.1186/s12913-024-10708-7 (PMC10949800; doi:10.1186/s12913-024-10708-7)
Supplement: Supplementary file 7 — Supplementary Material 7 [file 12913_2024_10708_MOESM7_ESM.docx]

**Supplementary Table 7.** The UNC occurence and reasons of the study based on The Unfinished Nursing Care Survey (=1) [36]

| PART A- Interventions Order | Cengia et al. [45] | |
| --- | --- | --- |
|  | **CS** | **RS** |
| Collect data about the patients’ care at the beginning of the shift, through the handover | 8^a^ | 5 |
| Perform a round at the beginning of the shift to know the patients, present themselves, and deepen their situation | 27 | 29 |
| Document properly the interventions provided and the revision of the care plan | 21 | 18 |
| Help patient in need in ambulation | 34 | 36 |
| Help patients who need it to get in the chair | 30 | 32 |
| Passive mobilization/changing position in bedrest patient | 33 | 35 |
| Helping patients who are unable to eat independently and/or have clinical problems (e.g., dysphagia) | 15 | 20 |
| Helping patients who are unable to drink independently and/or have clinical problems | 18 | 16 |
| To stimulate the patient to maintain/improve his/her independence | 24 | 25 |
| Provide personal hygiene to patients who need it | 4 | 9 |
| Provide mouth care to patients who need it | 32 | 34 |
| Perform physical assessment (e.g., skin integrity, and invasive device insertion site) | 12 | 14 |
| Check pressure ulcers and change dressing according to protocols | 9 | 12 |
| Perform bedside glucose monitoring as prescribed | 1 | 1 |
| Monitor intake/output | 5 | 6^d^ |
| Record vital signs as planned | 3 | 3 |
| Administer medications within 30 minutes of the time indicated in the prescription | 19 | 30^e^ |
| Administer PRN medications within 15 min from the patient's request | 13 | 11 |
| Monitor administered medications effects | 14 | 10 |
| Ensure patients' comfort (microclimate, patient positioning) | 22 | 26 |
| Monitor pain as planned | 7 | 6^d^ |
| Spend time with patients and their careres | 31 | 33 |
| Communicate with patients and careres | 29^c^ | 31 |
| Inform patients and their caregivers regarding the nursing care they are receiving | 25 | 24 |
| Emotionally support patients and careres by listening to their needs/concerns | 28 | 27 |
| Involve patients and careres in the discharge planning | 23 | 21 |
| Teach patients and careres how to self-care at home | 20 | 23 |
| Respond promptly to patients' calls (within 5 min) | 26^b^ | 28 |
| Go to the patients at the bedside without being called | 16 | 17 |
| Ensure intensive surveillance, reevaluating, those patients who are unstable or who present a risk of deteriorating conditions | 8^a^ | 8 |
| Prevent negative outcomes for patients at risk (e.g., falls, pressure ulcers, and malnutrition) | 11 | 13 |
| Prevent health care associated infections adopting good clinical practice (e.g., hand hygiene between patients, closed urinary drainage system) | 6 | 4 |
| Discuss with physicians and other staff members the problems and interventions needed by patients | 29^c^ | 22 |
| Supervise the tasks assigned to the nurse aides | 26^b^ | 30^e^ |
| Assess the effectiveness of the care provided, for example, reviewing if nursing care needs have been met | 22 | 19 |
| Fill in/update the clinical documentation/care plan in a comprehensive way | 17 | 15 |
| Perform clinical handover to adequately inform the next shift nursing team about patients' conditions | 2 | 2 |
| Provide clinical teaching to nursing students | 10 | 7 |
| PART B-Reasons for Unfinished Nursing Care Order |  | |
| Factor 1, Communication |  |  |
| Tension/conflicts within the nursing staff | 1 | 1 |
| Incomplete or interrupted communication among nursing staff | 7 | 9 |
| Tension/conflicts between nursing and medical staff | 10 | 8 |
| Incomplete or interrupted communication between nursing and medical staff | 12 | 12 |
| Lack of support/collaboration among team members | 11 | 11 |
| Factor 2, Priority setting |  |  |
| Inadequate nursing care model (e.g. functional task-oriented model of care) | 3 | 6 |
| Inaccurate initial priority setting | 2 | 3 |
| Inadequate priority re-assessment during the shift | 4 | 2 |
| Factor 3, Nurse Aides supervision |  |  |
| Nurse aides missed or delayed to report the tasks left undone | 5 | 7 |
| Inadequate supervision of the tasks assigned to the nurse aides | 6 | 4 |
| Incomplete or interrupted communication between nursing staff and nurse aides/assistive personnel | 9 | 5 |
| Factor 4, Material resources |  |  |
| Medications prescribed not available | 13 | 13 |
| Equipment not available/not functioning properly when needed | 14 | 14 |
| Other departments did not provide the service expected (e.g. delay in diagnostic processes) | 8 | 10 |
| Factor 5, Human resources |  |  |
| Inadequate number of nurses | 16 | 18 |
| Inadequate number of nurse aides | 15 | 15 |
| Factor 6, Work flow predictability |  |  |
| Unexpected rise in patient acuity | 17 | 17 |
| Heavy admission/discharge activity during the shift | 18 | 16 |

**Legend:** CS, Covid-19 Sample; RS, Reference Sample; *the order (e.g., 1,2,3) were determined according to the statistical values reported in the articles; ^a,b,c,d,e^, items with the same letter have emerged as equal within the article.
